# Associations of plasma NfL, GFAP, and t-tau with cerebral small vessel disease and incident dementia: longitudinal data of the AGES-Reykjavik Study

**DOI:** 10.1007/s11357-023-00888-1

**Published:** 2023-08-02

**Authors:** April C. E. van Gennip, Claudia L. Satizabal, Russell P. Tracy, Sigurdur Sigurdsson, Vilmundur Gudnason, Lenore J. Launer, Thomas T. van Sloten

**Affiliations:** 1 Department of Internal Medicine, Maastricht University Medical Centre, Maastricht, The Netherlands; 2 School for Cardiovascular Diseases (CARIM), Maastricht University, Maastricht, The Netherlands; 3 Glenn Biggs Institute for Alzheimer’s & Neurodegenerative Diseases, Department of Population Health Sciences, UT Health San Antonio, San Antonio, TX USA; 4 Laboratory for Clinical Biochemistry Research, The Robert Larner M.D. College of Medicine, University of Vermont, Burlington, VT USA; 5 Icelandic Heart Association, Kopavogur, Iceland; 6 Faculty of Medicine, University of Iceland, Reykjavik, Iceland; 7 Laboratory of Epidemiology and Population Sciences, National Institute On Aging, National Institutes of Health, Baltimore, MD USA; 8 Department of Vascular Medicine, Utrecht University Medical Center, Utrecht, The Netherlands

**Keywords:** Dementia, Cerebral small vessel disease, Plasma NfL, Plasma GFAP, Plasma t-tau, Epidemiology

## Abstract

We investigated the associations of plasma neurofilament light (NfL), glial fibrillary acidic protein (GFAP), and total tau (t-tau) with markers of cerebral small vessel disease (SVD) and with incident dementia. We also investigated whether associations of NfL, GFAP, and t-tau with incident dementia were explained by SVD. Data are from a random subsample (*n* = 1069) of the population-based AGES-Reykjavik Study who underwent brain MRI and in whom plasma NfL, GFAP, and t-tau were measured at baseline (76.1 ± 5.4 years/55.9% women/baseline 2002–2006/follow-up until 2015). A composite SVD burden score was calculated using white matter hyperintensity volume (WMHV), subcortical infarcts, cerebral microbleeds, and large perivascular spaces. Dementia was assessed in a 3-step process and adjudicated by specialists. Higher NfL was associated with a higher SVD burden score. Dementia occurred in 225 (21.0%) individuals. The SVD burden score significantly explained part of the association between NfL and incident dementia. WMHV mostly strongly contributed to the explained effect. GFAP was not associated with the SVD burden score, but was associated with WMHV, and WMHV significantly explained part of the association between GFAP and incident dementia. T-tau was associated with WMHV, but not with incident dementia. In conclusion, the marker most strongly related to SVD is plasma NfL, for which the association with WMHV appeared to explain part of its association with incident dementia. This study suggests that plasma NfL may reflect the contribution of co-morbid vascular disease to dementia. However, the magnitude of the explained effect was relatively small, and further research is required to investigate the clinical implications of this finding.

**Supplementary Information:**

The online version contains supplementary material available at 10.1007/s11357-023-00888-1.

## Introduction

Blood-based biomarkers for dementia risk will advance our ability to better understand the heterogeneous pathogenesis underlying dementia and its subtypes [1]. Neurofilament light (NfL), glial fibrillary acidic protein (GFAP), and total tau (t-tau) are promising fluid biomarkers with the potential for identifying pathological processes underlying dementia [1]. Brain NfL is a cytoskeletal component primarily of large myelinated axons that may reflect axonal damage [2, 3]; brain GFAP is an intermediate filament-III protein responsible for the cytoskeletal structure of astrocytes that is upregulated upon astrocyte activation [4, 5]; and brain t-tau is a microtubule-associated protein that regulates cytoskeletal dynamics of neurons that may reflect neurodegeneration [6]. The processes reflected by these fluid biomarkers, including neuronal damage, axon loss, demyelination, and astrogliosis (i.e., astrocyte activation), have also been associated with cerebral vascular damage [7, 8].

Higher plasma levels of NfL [9, 10] and GFAP [9, 11] have consistently been associated with a higher risk of all-cause dementia [10, 11] and Alzheimer’s disease dementia [9–11]. Some [9–12], but not all [10], studies have found an association between higher plasma t-tau levels and a higher risk of all-cause dementia [10, 12] or Alzheimer’s disease dementia [9, 10, 12]. The interpretation of higher circulating levels of NfL, GFAP, and t-tau, however, is still under investigation. Some population-based studies, but not all [13, 14], suggest that these biomarkers may be associated with underlying cerebral small vessel disease (SVD) [9, 15–18], which could either be co-morbid [19] or contributing to the dementia syndrome [20].

Here, we hypothesize that plasma levels of NfL, GFAP, and t-tau, rather than being causally related to SVD, reflect partly similar or overlapping mechanisms that play an important role in the pathogenesis of dementia. Therefore, we evaluated the associations of plasma NfL, GFAP, and t-tau to SVD markers, including white matter hyperintensity volume (WMHV), subcortical infarcts, cerebral microbleeds, and large perivascular spaces in a large population-based cohort. In addition, we investigated the question as to whether these plasma biomarkers are associated with the total burden of SVD, or individual SVD markers, and whether total or individual SVD burden explained the associations between the plasma biomarkers and incident dementia.

## Methods

### Study design

We used data from the Age, Gene/Environment Susceptibility (AGES)-Reykjavik Study. The AGES-Reykjavik Study is a longitudinal, population-based cohort study originating from the Reykjavik Study, as described previously in detail [21]. The Reykjavik Study was initiated in 1967 and included individuals born between 1907 and 1935 from the Reykjavik area. Between 2002 and 2006, 5764 randomly chosen surviving participants of the Reykjavik Study were examined for the AGES-Reykjavik Study. Among those participants with an MRI (*n* = 4811), a random sample of 1200 was selected for the measurement of NfL, GFAP, and t-tau as a part of the MarkVCID project [22]. Characteristics among individuals included in the substudy and those in the original cohort were comparable (Supplementary Table 1). The study was approved by the National Bioethics Committee in Iceland (approval number: VSN-00–063) and by the National Institute on Aging Intramural Institutional Review Board. All participants gave written informed consent.

### Plasma biomarkers NfL, GFAP, and t-tau

Fasting blood samples were collected and processed in accordance with established guidelines [23]. Plasma tubes were inverted 5 to 10 times and centrifuged for 10 min at 2000 × g within 1 h of collection. Five hundred microliter aliquots were transferred to polypropylene tubes and samples were places into − 80° freezer within 2 h of collection. Plasma samples were shipped to the Laboratory for Clinical Biochemistry Research at the University of Vermont, which has a strong quality assurance program for assays and is equipped with Simoa HD-1 Analzyer (Quanterix). Plasma levels of NfL, GFAP, and t-tau were measured using the Simoa Neurology 4-Plex Kit on a Simoa HD-1 Analyzer (Quanterix). Analytical ranges and inter-assay coefficients of variance are provided in Supplementary Table 2. A certified laboratory technician, blinded to diagnostic and ethnic groups, performed all assays between November and December 2019 using a single batch of reagents.

### Brain MRI measures

All eligible participants were offered high-resolution 1.5 T MRI (Signa Twin-Speed; General Electric Medical Systems). A standardized imaging protocol was used, as described previously [24, 25]. This protocol included the following sequences: 3-dimensional spoiled-gradient recalled T1-weighted, proton density/T2-weighted fast-spin echo, fluid-attenuated inversion recover (FLAIR), and T2a-weighted gradient-echo type echo-planer image (GRE-EPI). All images were acquired to give full brain coverage with slices angled parallel to the anterior commissure-posterior commissure line to give reproducible image views in the oblique-axial plane. We evaluated the following four markers of SVD: WMHV, subcortical infarcts, cerebral microbleeds, and large perivascular spaces. The identification of these markers was made in accordance with expert guidance that provided definitions and neuroimaging standards for markers and consequences of SVD [26]. Total brain parenchyma volume (TBV) and WMHV were computed automatically with a previously described image analysis pipeline [27] and were expressed as the percentage of total intracranial volume. Quality checks were done after tissue classification, as described in detail previously [27]. In brief, quality control consisted of visual inspection of a verification image for each subject including 14 a priori selected slice locations from each of the pulse sequences (T1, PD, T2, FLAIR), evenly distributed across the entire brain in the axial, coronal, and sagittal planes. Unsuccessful tissue classification that could not be rescued by repeated processing or manual editing occurred in 53 cases, mostly due to severe motion artifacts. These scans were excluded from the analytical sample. Other lesions were evaluated by trained radiographers using a standardized protocol [24, 25, 28]. Subcortical infarcts were defined as brain parenchyma defects not extending into the cortex, with a minimum diameter of 4 mm and a signal intensity equal to cerebrospinal fluid on all pulse sequences (T2-weighted, proton density–weighted, and FLAIR), and surrounded by an area of high intensity on FLAIR images and without evidence of hemosiderin on T2a-weighted GRE-EPI sequence [25]. Cerebral microbleeds were defined as focal areas of signal void visible on the T2a-weighted GRE-EPI sequence [24]. Large perivascular spaces were defined as defects on the subcortical area without a rim or area of high signal intensity on FLAIR and without evidence of hemosiderin on the T2a-weighted GRE-EPI sequence [28]. The total number of large perivascular spaces was based on the presence in the basal ganglia complex, along the paths of the perforating lenticulostriate arteries, and in white matter along the paths of the perforating medullary arteries [28]. Information on reproducibility of the process, including the image acquisition and the automatic pipeline, is provided in detail elsewhere [27]. For the volumetric markers, reproducibility was performed in 32 subjects and yielded an interclass correlation 0.98 for both TBV and WMHV [27]. For the other markers, intra- and inter-observer reliability was based on 2 ratings within a 6-month interval and indicated good reliability. Intra-observer reliability was 0.89 and 0.93 for subcortical infarcts [29], 0.75 and 0.73 for cerebral microbleeds [30], and 0.88 and 0.93 for large perivascular spaces [28], respectively. Inter-observer reliability was 0.76 for subcortical infarcts [29], 0.70 for cerebral microbleeds [30], and 0.66 for large perivascular spaces, respectively [28].

### Incident all-cause dementia

Incident all-cause dementia was assessed at the follow-up examination (2007–2011) using a 3-step procedure, as described previously [31]. This was the same assessment used for the ascertainment of prevalent all-cause dementia at the baseline examination performed by the same panel of professionals [32]. In brief, the Mini-Mental State Examination and the Digit Symbol Substitution Test were administered to all participants. Individuals who screened positive based on a combination of these tests (< 24 on the Mini-Mental State Examination or < 8 on the Digit Symbol Substitution Test) were administered a diagnostic battery of neuropsychological tests. Based on performance on the Trails B and the Rey Auditory Verbal Learning Test, a subset of these individuals (Auditory Verbal Learning test ≤ 18 or Trails B ≥ 8 for the ratio of time taken for Trails B/Trails A corrected for the number correct: (time trails B/number correct Trails B)/(time Trails A/number correct Trails A)) underwent a proxy interview and were examined by a neurologist. A consensus diagnosis, based on the Diagnostic and Statistical Manual of Mental Disorders 4^th^ edition criteria, was made by a panel of experts including a geriatrician, a neurologist, a neuropsychologist, and a neuroradiologist. In addition, all participants were continuously followed up for dementia diagnosis through vital statistics, hospital records, and the nursing and home-based Resident Assessment Instrument [33]. Follow-up for dementia ended October 4, 2015.

### Covariates

Education level (primary, secondary, and college/university) and smoking history (never, former, current) were assessed by questionnaire. Medication use was assessed by questionnaire and from medication bottles brought to the clinic. Blood pressure, body mass index, and lipid levels were measured using standardized protocols [21]. We defined diabetes as a self-reported history of diabetes, use of blood glucose–lowering drugs, or a fasting blood glucose level of ≥ 7.0 mmol/l. Stroke (i.e., symptomatic brain infarct or hemorrhage) prevalent at baseline was obtained from medical records. Incident strokes that occurred between the baseline and follow-up examination were adjudicated by a dementia neurologist, a stroke neurologist, and a neuroradiologist.

### Analytical sample

Of the 1200 individuals in this biomarker substudy, 6 participants had missing data on one or more of the plasma biomarkers and another 8 did not have specific MRI images needed for assessment of cerebral microbleeds. Missing data on plasma biomarkers was due to technical reasons, including missing sample (*n* = 1), insufficient volume available (*n* = 3), and invalid result (*n* = 2). In addition, we excluded 10 participants with missing data on covariates. In the remaining 1176 participants, 107 were excluded because of a diagnosis of dementia at baseline (*n* = 47) or because of missing data on incident dementia (*n* = 60). The final study sample included 1069 participants (Supplementary Fig. 1). Participants excluded from the biomarker substudy sample were older, less educated, and were more likely to have hypertension or type 2 diabetes compared to those included in the analysis (Supplementary Table 3).

### Statistical analysis

We summarized the four markers of SVD into a composite sum score (range 0–4) to reflect burden of SVD (SVD burden score) as done previously [34]. One point per SVD marker was assigned based on the following cut-offs: for WMHV highest quartile versus lowest three quartiles; and for subcortical infarcts, cerebral microbleeds, and large perivascular spaces, presence (i.e., ≥ 1 lesion(s)) versus absence). In all analyses, the SVD burden score was analyzed on a continuous scale to enhance the statistical power of our analysis, as done previously [34, 35]. We also evaluated the SVD burden score on an ordinal scale. Plasma biomarkers were transformed using a natural logarithm (i.e., base-e log) to normalize their skewed distribution.

The statistical analysis proceeded in three stages. First, to evaluate the relation between plasma biomarkers and the SVD burden score, we used linear regression to estimate regression coefficients (betas) and 95% confidence intervals (95%CIs) for the association of plasma NfL, GFAP, and t-tau with the SVD burden score. Second, to evaluate the association between plasma biomarkers and incident dementia, we used Cox regression to estimate hazard ratios (HRs) and 95% CIs for the association of plasma NfL, GFAP, and t-tau with incident dementia using time-in-study as the time scale. Follow-up time was calculated from the AGES-Reykjavik baseline examination (2002–2006) to incidence of dementia, death, or end of follow-up (October 4, 2015), whichever came first. The proportional hazard assumption was assessed by visual inspection of Kaplan–Meier curves (Supplementary Fig. 2). Third, to investigate whether the SVD burden score explained the association of plasma NfL, GFAP, and t-tau with incident dementia (if any), we entered the SVD burden score as a covariate in the biomarker-dementia models. We did not consider the SVD burden score to be on the putative causal pathway of the plasma biomarkers leading to dementia, and we, therefore, did not do a formal mediation analysis. To quantify the degree to which the SVD burden score attenuated the association of plasma biomarkers with incident dementia, we calculated the explained effects. The explained effects were calculated as the multiplied effects of plasma biomarkers and brain MRI markers and brain MRI markers and incident dementia, adjusted for the plasma biomarkers [36]. The calculation of the explained effect is summarized in Supplementary Fig. 3. We used bootstrapping (10,000 samples) to calculate bias-corrected 95% CIs for the explained effects.

All analyses were adjusted for age and sex (model 1) and additionally for education level, smoking history, diabetes status, body mass index, total cholesterol-to-HDL cholesterol ratio, use of lipid-modifying medication, systolic blood pressure, and use of antihypertensive medication (model 2). These covariates were selected on the basis of their biological plausibility, since they are known to be associated with SVD [37] or dementia [38]. Data on the association between plasma NfL, GFAP, and t-tau and the covariates included in model 2 is still limited [39]. However, these covariates are known to be associated with the neurodegenerative mechanisms that are presumed to be reflected by plasma levels of NfL, GFAP, and t-tau [40, 41].

We performed several sensitivity analyses. First, we repeated the analyses for each of the individual SVD markers separately. Second, to minimize potential confounding or mediating effects by TBV or stroke, we repeated the analysis additionally adjusting for TBV and baseline stroke or incident stroke during follow-up. Third, to investigate the effect of the definition of WMHV, we evaluated WMHV expressed on a continuous scale and WMHV expressed as higher versus lower than the median.

## Results

The mean age of the participants at baseline was 76.1 (SD: 5.4) and 55.9% were female. Overall, 21.0% of the participants developed incident dementia after a mean follow-up of 8.7 (SD: 3.5) years. Table 1 shows the characteristics of the study population and by tertiles of plasma NfL. Characteristics by tertiles of plasma GFAP and t-tau are provided in the Supplementary Material (Supplementary Tables 4 and 5). In general, participants with the highest compared to the lowest two tertiles of plasma NfL were older, more often female, had a worse cardiovascular risk profile and were more likely to have a stroke (Table 1). For instance, there was an increase in age from the lowest tertile to the highest tertile of plasma NfL.

**Table 1:** Characteristics of the total study population, and according to tertiles of plasma NfL

Characteristics	Total study population (*n* = 1069)	Tertiles of plasma NfL		
		Lowest tertile (*n* = 357, 33.4%)	Middle tertile (*n* = 355, 33.2%)	Highest tertile (*n* = 357, 33.4%)
Age at baseline, years	76.1 (5.4)	73.4 (4.3)	75.8 (5.1)	79.2 (5.3)
Female, No (%)	598 (55.9)	195 (54.6)	197 (55.5)	206 (57.7)
Education level				
Primary, No (%)	244 (22.8)	71 (19.9)	80 (22.5)	93 (26.1)
Secondary, No (%)	523 (48.9)	194 (54.3)	172 (48.5)	157 (44.0)
College/university, No (%)	302 (28.3)	92 (25.8)	103 (29.0)	107 (30.0)
Smoking history				
Never smoker, No (%)	445 (41.6)	132 (37.0)	151 (42.5)	162 (45.4)
Former smoker, No (%)	487 (45.6)	173 (48.5)	157 (44.2)	157 (44.0)
Current smoker, No (%)	137 (12.8)	52 (14.6)	47 (13.2)	38 (10.6)
Type 2 diabetes, No (%)	109 (10.2)	34 (9.5)	37 (10.4)	38 (10.6)
Hypertension, No (%)	866 (81.0)	279 (78.2)	281 (79.2)	206 (85.7)
Stroke				
Baseline, No (%)	49 (4.6)	7 (2.0)	13 (3.9)	28 (7.8)
Incident, No (%)	96 (9.0)	21 (5.9)	28 (7.9)	47 (13.2)
Body mass index, kg/m^2^	27.0 (4.4)	27.9 (4.3)	27.2 (4.4)	26.0 (4.4)
Systolic blood pressure, mmHg	142.2 (20.1)	141.3 (19.8)	139.8 (19.3)	145.7 (20.8)
Diastolic blood pressure, mmHg	74.0 (10.0)	75.9 (9.7)	73.6 (9.6)	72.6 (10.4)
Total-to-HDL cholesterol ratio	3.8 (1.1)	3.9 (1.1)	3.8 (1.1)	3.6 (1.1)
Lipid-modifying medication, No (%)	244 (22.8)	77 (21.6)	85 (23.9)	82 (23.0)
Antihypertensive medication, No (%)	683 (63.9)	202 (56.6)	224 (63.1)	257 (72.0)
Incident dementia, No (%)	225 (21.0)	44 (12.3)	74 (20.8)	107 (30.0)
TBV^a^, %	72.2 (3.8)	73.3 (3.6)	72.2 (3.6)	71.1 (3.7)
SVD burden score^b^	0.7 (0.8)	0.5 (0.7)	0.6 (0.8)	0.9 (1.0)
WMHV^a^, %	0.9 (0.5; 1.7)	0.7 (0.4; 1.2)	0.8 (0.5; 1.4)	1.3 (0.7; 2.3)
Highest quartile of WMHV, No (%)	262 (24.5)	56 (15.7)	68 (19.2)	138 (38.7)
Subcortical infarcts, No (%)	131 (12.3)	31 (8.7)	36 (10.1)	64 (17.9)
Cerebral microbleeds, No (%)	123 (11.5)	34 (9.5)	32 (9.0)	57 (16.0)
Large perivascular spaces, No (%)	191 (17.9)	52 (14.6)	68 (19.2)	71 (19.9)
Plasma NfL, pg/ml	22.1 (16.8; 29.9)	14.9 (13.0; 16.8)	22.1 (20.0; 24.2)	34.1 (29.9; 42.2)
Plasma GFAP, pg/ml	176.8 (130.1; 233.1)	134.4 (105.7; 174.3)	176.7 (140.3; 222.8)	228.9 (180.7; 348.3)
Plasma t-tau, pg/ml	2.61 (1.97; 3.44)	2.37 (1.77; 3.09)	2.57 (1.97; 3.29)	3.16 (2.23; 4.03)

Data are means (standard deviation) or median (interquartile range)

Abbreviations: *NfL*, neurofilament light; *HDL*, high-density lipoprotein; *TBV*, total brain volume; *SVD*, cerebral small vessel disease; *WMHV*, white matter hyperintensity volume; *GFAP*, glial fibrillary acidic protein; *t-tau*, total tau

^a^TBV and WMHV were expressed as percentage of intracranial volume

^b^SVD burden score was calculated by assigning one point per cerebral small vessel disease marker based on the following cut-offs (range 0–4): for WMHV highest quartile vs lowest three quartiles, and for subcortical infarcts, cerebral microbleeds, and large perivascular spaces presence vs absence

**Fig. 1:**
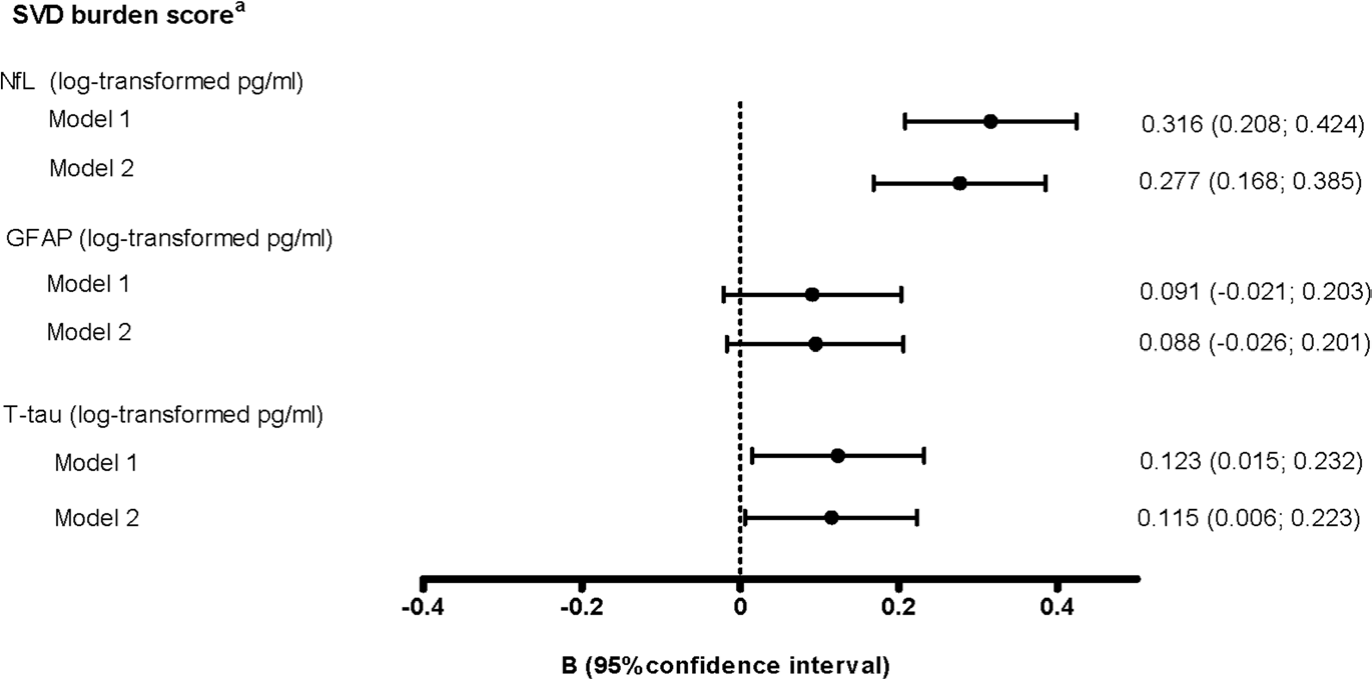
Associations between plasma NfL, GFAP, and t-tau and the SVD burden score^a^. Betas are expressed per natural log-transformed pg/ml higher plasma NfL, GFAP, or t-tau. Model 1 adjusted for age and sex. Model 2 additionally adjusted for education level, diabetes status, smoking history, body mass index, total cholesterol-to-HDL cholesterol ratio, use of lipid-modifying medication, systolic blood pressure, and use of antihypertensive medication. Abbreviations: SVD, cerebral small vessel disease; NfL, neurofilament light; GFAP, glial fibrillary acidic protein; t-tau, total tau. ^a^SVD burden score was calculated by assigning one point per cerebral small vessel disease marker based on the following cut-offs (range 0–4): WMHV highest quartile vs lowest three quartiles, and for subcortical infarcts, cerebral microbleeds, and large perivascular spaces presence vs absence

**Fig. 2:**
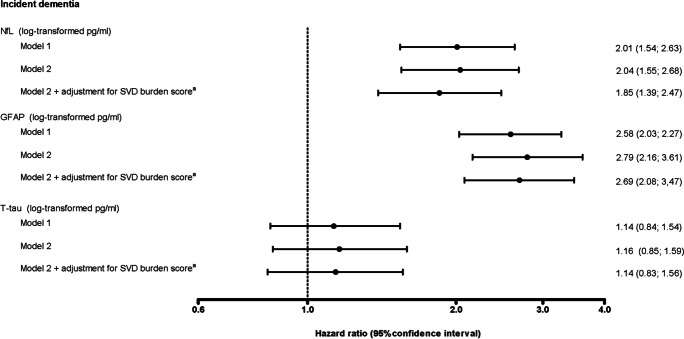
Associations between plasma NfL, GFAP, and t-tau and incident dementia with and without adjustment for the SVD burden score^a^. Hazard ratios for incident dementia are expressed per natural log-transformed pg/ml higher plasma NfL, GFAP, or t-tau. Model 1 adjusted for age and sex. Model 2 additionally adjusted for education level, diabetes status, smoking history, body mass index, total cholesterol-to-HDL cholesterol ratio, use of lipid-modifying medication, systolic blood pressure, and use of antihypertensive medication. Models 1 and 2 represent the total effect, and model 2 + adjustment for the SVD burden score represents the direct effect. Total and direct effect are defined in Supplementary Fig. 3. Abbreviations: NfL, neurofilament light; GFAP, glial fibrillary acidic protein; t-tau, total tau; SVD, cerebral small vessel disease. ^a^SVD burden score was calculated by assigning one point per cerebral small vessel disease marker based on the following cut-offs (range 0–4): WMHV highest quartile vs lowest three quartiles, and for subcortical infarcts, cerebral microbleeds, and large perivascular spaces presence vs absence

A higher plasma NfL and a higher t-tau were associated with a higher SVD burden score (Fig. 1). A higher plasma NfL and GFAP were associated with a higher risk of dementia after adjustment for potential confounders (Fig. 2, model 2).

When we additionally adjusted the association between plasma NfL and incident dementia for the SVD burden score, the association attenuated but remained statistically significant (Fig. 2, model 3). The SVD burden score statistically significantly explained part of the association between plasma NfL and dementia (HR 1.07 (95% CI 1.02; 1.15)) (Table 2). The association between plasma GFAP and incident dementia was not explained by the SVD burden score (Fig. 2, model 3, and Table 2).

**Table 2:** Total effects, direct effects, and explained effects by the SVD burden score^a^ of the associations between plasma NfL, GFAP, and t-tau and incident dementia

Plasma biomarker	Total effects	Direct effects	Explained effects
	Hazard ratio (95% confidence interval)		
NfL	2.04 (1.55; 2.68)	1.85 (1.39; 2.47)	1.07 (1.02; 1.15)
GFAP	2.79 (2.16; 3.61)	1.69 (2.08; 3.47)	1.03 (0.99; 1.07)
T-tau	1.16 (0.85; 1.59)	1.14 (0.83; 1.56)	1.04 (1.00; 1.09)

Hazard ratios for incident dementia are expressed per natural log-transformed pg/ml higher plasma NfL, GFAP, or t-tau. Total effect, direct effect, and explained effect are defined in Supplementary Fig. 3. The explained effect quantifies the degree to which the SVD burden score^a^ attenuated the association of plasma biomarkers with incident dementia. All analyses adjusted for age, sex, education level, diabetes status, smoking history, body mass index, total cholesterol-to-HDL cholesterol ratio, use of lipid-modifying medication, systolic blood pressure, and use of antihypertensive medication

Abbreviations: *SVD*, cerebral small vessel disease; *NfL*, neurofilament light; *GFAP*, glial fibrillary acidic protein; *t-tau*, total tau

^a^SVD burden score was calculated by assigning one point per cerebral small vessel disease marker based on the following cut-offs (range 0–4): WMHV highest quartile vs lowest three quartiles, and for subcortical infarcts, cerebral microbleeds, and large perivascular spaces presence vs absence

### Sensitivity analysis

The analyses with the SVD burden score modelled on an ordinal scale showed a linear increase for the risk of dementia for a higher SVD burden score (Supplementary Table 6). In addition, plasma NfL, but not GFAP and t-tau, increased linearly for a higher SVD burden score (Supplementary Table 7). The non-linear association between plasma GFAP and t-tau and the SVD burden score is in accordance with the non-significant finding of the explained effect by the SVD burden score of the associations between plasma GFAP and t-tau and incident dementia. Additionally adjusting the association between the plasma biomarkers and incident dementia for the SVD burden score on an ordinal scale yielded results similar to those obtained when we additionally adjusted for the SVD burden score on a continuous scale (Supplementary Fig. 4). A higher plasma NfL was associated with a higher WMHV and presence of subcortical infarcts; only WMHV explained part of the association of plasma NfL with incident dementia (Supplementary Fig. 5 and 6 and Supplementary Table 8). A higher plasma GFAP was only associated with WMHV, and similar to plasma NfL, WMHV explained part of the association of plasma GFAP with dementia (Supplementary Figs. 5 and 6 and Supplementary Table 8). Plasma t-tau was associated with a higher WMHV, but not with any of the other markers of SVD (Supplementary Fig. 5). Results were similar when we additionally adjusted for TBV or prevalent or incident stroke (Supplementary Fig. 7 to 10 and Supplementary Table 9 and 10). Results were similar using WMHV on a continuous scale, or using WMHV expressed as higher versus lower than the median, instead of comparing those in the highest quartile of WMHV to those in the lowest three quartiles (Supplementary Figs. 11 and 12 and Supplementary Tables 11).

## Discussion

In this study, a higher plasma NfL was associated with a higher total SVD burden score. The SVD burden score statistically significantly explained part of the association between plasma NfL and incident dementia. Of the different components of the SVD burden score, WMHV was the strongest component as it drove the attenuation of the association between NfL and incident dementia. Plasma GFAP was not associated with the SVD burden score, but was associated with WMHV, which significantly explained part of the association between GFAP and incident dementia. Plasma t-tau was not linearly associated with a higher SVD burden score, but was associated with WMHV. Plasma t-tau was not associated with incident dementia. Together these results suggest that these plasma biomarkers are differentially associated with markers of SVD and burden of SVD. The biomarker most strongly related to SVD is plasma NfL whose association with WMHV appeared to partly explain its association with incident dementia. Our study also suggests that “burden of SVD” is less important as an explaining factor, as having subcortical infarcts, cerebral microbleeds, and large perivascular spaces did not contribute significantly to the NfL–dementia association.

Most data on SVD comes from studies on WMHV. With the exception of one study [16] (*n* = 1362), these studies were relatively small (*n* < 300) [15–17] or did not adjust for cardiovascular risk factors [9, 13, 15, 17]. Consistent with our study findings, most of these studies (including the largest study [16]), but not all [9, 13], found that WMHV was associated with a higher plasma NfL [9, 15, 16], GFAP [16, 17], but not with t-tau [9, 13, 16]. Consistent with our findings, cerebral microbleeds measured in 3 community-based studies of individuals with an average age around 75 year and with sample sizes *n* = 712 or less were not associated with plasma NfL [14, 15], GFAP [17], or t-tau [14]. One large study including 3680 individuals with an average age of 55 years [18] did find a significant association of t-tau with cerebral microbleeds. The reasons for this inconsistent finding may reflect the younger age of that cohort compared to the other cohorts, the larger sample size or differences in MRI sequences. We extend the results of these previous studies in several ways: we compared and took account of multiple vascular lesions in the brain and examined the contribution of the burden of SVD to the association of plasma NfL, GFAP, and t-tau with incident dementia.

This study suggests that plasma levels of NfL, but not GFAP, may reflect the contribution of co-morbid vascular disease in the brain to dementia. Plasma GFAP may potentially be related to other mechanisms that have direct neurotoxic effects that were not evaluated in the present study, including amyloid pathology [42, 43]. Activated astrocytes, with high expression of GFAP, are found to surround amyloid plaques in Alzheimer’s disease [5]. Other mechanisms that might play a role are large vessel disease [44] and oxidative stress [45]. Additionally, our results suggest that plasma NfL, GFAP, and t-tau are differentially associated with markers of SVD. Possibly, these plasma biomarkers may be more likely to reflect diffuse cerebral damage due to white matter hyperintensities as compared to the focal damage due to subcortical infarcts, cerebral microbleeds, and large perivascular spaces [8]. However, this study is the first to explore multiple vascular lesions in the brain in relation to plasma NfL, GFAP, and t-tau, and the specific clinical consequences of the SVD lesion types are not fully understood. In addition, the magnitude of the explained effects was relatively small, and therefore, the clinical implications of these biomarkers in identifying the pathological processes underlying dementia are unclear. Further study is needed to clarify these issues.

In this study, plasma t-tau was associated with WMHV, but we did not observe an association between plasma t-tau and incident dementia. This may suggest that plasma t-tau may be a marker of WMHV, but that it is not specific to the neuropathology underlying dementia. The role of plasma t-tau as a biomarker of dementia risk is less clearly established than those of plasma NfL and GFAP. Consistent with our study findings, a previous population-based study [10] that included 4444 individuals did not find an association between plasma t-tau and incident dementia. In contrast, two other studies (*n* = 1453 [12] and *n* = 1327 [9]) found an association between a higher plasma t-tau and a higher risk of dementia. The reasons for these inconsistent findings are not fully clear, but may be due to differences in adjustment for potential confounders (adjustment for sociodemographic factors only [9] vs extensive adjustment for sociodemographic and cardiovascular risk factors [10, 12]). Furthermore, it is possible that plasma levels of t-tau may be less useful as markers for dementia risk because it may not accurately reflect levels in the cerebrospinal fluid, as suggested previously [46, 47]. This may be due to peripheral degradation of t-tau into undetectable fragments [46], or, alternatively, secretion of t-tau in other organs than the brain, including the kidney and skeletal muscle [47].

Key strengths of this study include the large population-based sample, the comprehensive assessment of multiple biomarkers measured in plasma and on brain MRI, and the extensive characterization of participants, which enabled us to adjust for a series of potential confounders.

This study has several limitations. First, the plasma biomarkers NfL, GFAP, and t-tau were measured at the baseline examination only, and, therefore, we cannot investigate the temporality of levels of these plasma biomarkers. Possibly, accumulative data about the plasma levels of these biomarkers across the life course may be a stronger determinant of dementia risk, and may be more strongly related to the total burden of SVD. Second, although we adjusted for a large series of potential confounders, we cannot exclude the possibility of residual confounding. For example, it is possible that SVD explains part of the association between plasma NfL and incident dementia because plasma levels of NfL are also known to increase with normal aging [3]. Third, some of the biomarkers investigated may be more strongly related to pathology specific to dementia subtypes (i.e., Alzheimer’s disease dementia or vascular dementia). However, we did not investigate associations with specific dementia type, and this requires further study. In addition, plasma phosphorylated tau (p-tau) and amyloid-beta may be more specific to the brain pathology in dementia as compared to plasma NfL, GFAP, and t-tau [6], but these biomarkers were not available in the present study. Fourth, individuals excluded in the present study due to missing data were older, less educated, and had a worse cardiovascular risk profile compared to those included in the analysis. This may have led to an underestimation of the reported findings due to lower variation in biomarkers and lower incidence of dementia. Fifth, the study population consisted mostly of Caucasian individuals, and the results may therefore not apply to other ethnic groups. Sixth, in this study, some cases of incident dementia were identified through medical records and vital statistics. We cannot exclude the possibility that this approach had a lower sensitivity and specificity compared to the 3-step procedure used during the clinical baseline and follow-up examination and the nursing and home-based Resident Assessment Instrument.

In conclusion, the plasma biomarker most strongly related to SVD is NfL whose association with WMHV appeared to partly explain its association with incident dementia. This study suggests that plasma NfL may reflect the contribution to dementia of co-morbid vascular disease, particularly of WMHV. However, the magnitude of the explained effect was relatively small, and further research is required to investigate the clinical implications of these findings.

### Supplementary Information

Below is the link to the electronic supplementary material.

Supplementary file1 (PDF 1.54 MB)

## Data Availability

The data that support the findings of this study are available from the corresponding author upon reasonable request.
