# Supplementary material for: Associations of plasma NfL, GFAP, and t-tau with cerebral small vessel disease and incident dementia: longitudinal data of the AGES-Reykjavik Study
Source: GeroScience. 2023 Aug 2;46(1):505–16. doi: 10.1007/s11357-023-00888-1 (PMC10828267; doi:10.1007/s11357-023-00888-1)
Supplement: Supplementary file 1 — Supplementary file1 (PDF 1.54 MB) [file 11357_2023_888_MOESM1_ESM.pdf]

Associations of plasma NfL, GFAP, and t-tau with cerebral small vessel disease and incident dementia: longitudinal data of the AGES-Reykjavik Study

April CE van Gennip, Claudia L Satizabal, Russell P Tracy, Sigurdur Sigurdsson, Vilmundur Gudnason, Lenore J Launer, Thomas T van Sloten

Corresponding author: Thomas T. van Sloten, Department of Vascular Medicine, University Medical Center Utrecht, Utrecht 3508 GA, the Netherlands

Phone +31621214590 | e-mail: t.t.vansloten@umcutrecht.nl

GeroScience

Supplemental Material

Supplemental Tables

Supplementary Table 1. Characteristics of participants included in the biomarker substudy, and those included in the original AGES-Reykjavik cohort

Supplementary Table 2. Analytical ranges and inter-assay coefficient of variation of the plasma biomarkers

Supplementary Table 3. Characteristics of included study participants and excluded individuals

Supplementary Table 4. Characteristics of the total study population, and according to tertiles of plasma GFAP

Supplementary Table 5. Characteristics of the total study population, and according to tertiles of plasma t-tau

Supplementary Table 6. Association between the SVD burden score[a] on an ordinal scale and incident dementia

Supplementary Table 7. Association between plasma NfL, GFAP, and t-tau and the SVD burden score[a] on an ordinal scale

Supplementary Table 8. Total effects, direct effects, and explained effects by WMHV[a], subcortical infarcts, cerebral microbleeds, and large perivascular spaces of the associations between plasma NfL, GFAP, and t-tau and incident dementia

Supplementary Table 9. Total effects, direct effects, and explained effects by the SVD burden score[a] of the associations between plasma NfL, GFAP, and t-tau and incident dementia – additional adjustment for TBV[b] and prevalent or incident stroke

Supplementary Table 10. Total effects, direct effects, and explained effects by WMHV[b], subcortical infarcts, cerebral microbleeds, and large perivascular spaces of the associations between plasma NfL, GFAP, and t-tau and incident dementia – additional adjustment for TBV[a] and prevalent or incident stroke

Supplementary Table 11. Total effects, direct effects, and explained effects by WMHV[a] on a continuous scale and expressed as higher versus lower than the median of the associations between plasma NfL, GFAP, and t-tau and incident dementia

Supplemental Figures

Supplementary Figure 1. Flow chart derivation of the study population

Supplementary Figure 2. Survival curves for incident dementia according to tertiles of plasma NfL (Panel a), GFAP (Panel b), and t-tau (Panel c)

Supplementary Figure 3. Summary of the statistical analysis

Supplementary Figure 4. Associations between plasma NfL, GFAP, and t-tau and incident dementia with and without adjustment for the SVD burden score[a] on an ordinal scale

Supplementary Figure 5. Associations between on the one hand plasma NfL, GFAP, and t-tau and on the other WMHV[a] (Panel a), subcortical infarcts (Panel b), cerebral microbleeds (Panel c) and large perivascular spaces (Panel d)

Supplementary Figure 6. Associations between plasma NfL, GFAP, and t-tau and incident dementia with and without adjustment for WMHV[a], subcortical infarcts, cerebral microbleeds, and large perivascular spaces

Supplementary Figure 7. Associations between plasma NfL, GFAP, and t-tau and the SVD burden score[a] - additional adjustment for TBV[b] and prevalent or incident stroke

Supplementary Figure 8. Associations between plasma NfL, GFAP, and t-tau and incident dementia with and without adjustment for the SVD burden score[a] - additional adjustment for TBV[b] (Panel a) and prevalent and incident stroke (Panel b)

Supplementary Figure 9. Associations between on the one hand plasma NfL, GFAP, and t-tau and on the other WMHV[b] (Panel a), subcortical infarcts (Panel b), cerebral microbleeds (Panel c) and large perivascular spaces (Panel d) - additional adjustment for TBV[a] and prevalent or incident stroke

Supplementary Figure 10. Associations between plasma NfL, GFAP, and t-tau and incident dementia with and without adjustment for WMHV[b], subcortical infarcts, cerebral microbleeds, and large perivascular spaces - additional adjustment for TBV[a] (Panel a) and prevalent and incident stroke (Panel b)

Supplementary Figure 11. Associations between on the one hand plasma NfL, GFAP, and t-tau and on the other WMHV[a] on a continuous scale (Panel a), and expressed as higher versus lower than the median (Panel b)

Supplementary Figure 12. Associations between plasma NfL, GFAP, and t-tau and incident dementia with and without adjustment for WMHV[a] on a continuous scale (Panel a), and expressed as higher versus lower than the median (Panel b)

Supplementary Table 1. Characteristics of participants included in the biomarker substudy and those included in the original AGES-Reykjavik cohort

Characteristics	Biomarker substudy (n=1,200)	Original cohort (n=5,764)
Age at baseline, years	76.3 (5.5)	77.0 (5.9)
Female, No (%)	677 (56.4)	3326 (57.7)
Education level[a]		
- Primary, No (%)	287 (24.1)	1264 (23.7)
- Secondary, No (%)	582 (48.9)	2654 (49.8)
- College/university, No (%)	322 (27.0)	1413 (26.5)
Smoking history[b]		
- Never smoker, No (%)	504 (42.0)	2468 (44.3)
- Former smoker, No (%)	540 (45.0)	2418 (43.4)
- Current smoker, No (%)	155 (12.9)	683 (12.3)
Type 2 diabetes, No (%)	127 (10.6)	749 (13.0)
Hypertension[c], No (%)	982 (81.8)	4638 (81.1)
Stroke		
- Baseline, No (%)	60 (5.0)	334 (5.8)
- Incident, No (%)	106 (8.8)	494 (8.6)
Body mass index[d], kg/m^2	27.0 (4.5)	27.0 (4.4)
Systolic blood pressure[e], mmHg	142.3 (20.1)	142.4 (20.8)
Diastolic blood pressure[e], mmHg	73.9 (9.9)	73.8 (9.8)
Total-to-HDL cholesterol ratio[f]	3.6 (3.0; 4.4)	3.6 (2.9; 4.4)
Lipid-modifying medication, No (%)	272 (22.7)	1251 (21.7)
Antihypertensive medication[g], No (%)	773 (64.4)	3662 (64.0)
Incident dementia[h], No (%)	228 (19.8)	1099 (20.2)
TBV[i], %	72.1 (4.1)	73.0 (3.7)
SVD burden score[j]	0.7 (0.9)	0.7 (0.9)
WMHV[k], %	0.9 (0.5; 1.7)	0.8 (0.5; 1.4)
Highest quartile of WMHV[k], No (%)	301 (25.1)	647 (25.0)
Subcortical infarcts[l], No (%)	148 (12.3)	202 (7.6)
Cerebral microbleeds[m], No (%)	145 (12.2)	456 (17.1)
Large perivascular spaces[l], No (%)	222 (18.5)	432 (16.2)

Data are means (standard deviation) or median (interquartile range).
Abbreviations: HDL, high-density lipoprotein; TBV, total brain volume; SVD, cerebral small vessel disease; WMHV, white matter hyperintensity volume.
[a] Data available in n=1191 in the biomarker substudy and n=5331 in the original cohort, respectively. [b] Data available in n=1199 in the biomarker substudy and n=5569 in the original cohort, respectively. [c] Data available in n=1200 in the biomarker substudy and in n=5717 in the original cohort, respectively. [d] Data available in the biomarker substudy in n=1199 and n=5696 in the original cohort, respectively. [e] Data available in 1200 in the biomarker substudy and n=5717 in the original cohort, respectively. [f] Data available in 1200 in the biomarker substudy and n=5760 in the original cohort, respectively. [g] Data available in 1200 in the biomarker substudy and n=5718 in the original cohort, respectively. [h] Data available in 1152 in the biomarker substudy and n=5452 in the original cohort, respectively. [l] TBV was expressed as percentage of intracranial volume. Data available in 1200 in the biomarker substudy and n=2588 in the original cohort, respectively. [j] SVD burden score was calculated by assigning one point per cerebral small vessel disease marker based on the following cut-offs (range 0-4): WMHV highest quartile vs lowest three quartiles, and for subcortical infarcts, cerebral microbleeds, and large perivascular spaces presence vs absence. Data available in 1192 in the biomarker substudy and n=2588 in the original cohort, respectively. [k] WMHV was expressed as percentage of intracranial volume. Data available in 1200 in the biomarker substudy and n=2588 in the original cohort, respectively. [l] Data available in 1200 in the biomarker substudy and n=2672 in the original cohort, respectively. [m] Data available in 1192 in the biomarker substudy and n=2588 in the original cohort, respectively.

Supplementary Table 2. Analytical ranges and inter-assay coefficient of variation of the plasma biomarkers

Plasma biomarker	Analytical range (pg/ml)	Inter-Assay Coefficient of Variance, %
NfL	0.4 - 1,504	13.2
GFAP	0.8 - 3,420	13.9
T-tau	0.09 - 245	19.4

Abbreviations: NfL, neurofilament light; GFAP, glial fibrillary acidic protein; t-tau, total tau.

Supplementary Table 3. Characteristics of included study participants and excluded individuals

Characteristics	Included individuals (n=1,069)	Excluded individuals (n=131)
Age at baseline, years	76.1 (5.4)	78.1 (6.1)
Female, No (%)	598 (55.9)	79 (60.3)
Education level		
- Primary, No (%)	244 (22.8)	43 (35.2)
- Secondary, No (%)	523 (48.9)	59 (48.4)
- College/university, No (%)	302 (28.3)	20 (16.4)
Smoking history		
- Never smoker, No (%)	445 (41.6)	59 (45.0)
- Former smoker, No (%)	487 (45.6)	53 (40.5)
- Current smoker, No (%)	137 (12.8)	18 (13.7)
Type 2 diabetes, No (%)	109 (10.2)	18 (13.7)
Hypertension, No (%)	866 (81.0)	116 (88.5)
Stroke		
- Baseline, No (%)	49 (4.6)	11 (8.4)
- Incident, No (%)	96 (9.0)	10 (7.6)
Body mass index, kg/m^2	27.0 (4.4)	26.2 (4.6)
Systolic blood pressure, mmHg	142.2 (20.1)	143.2 (19.8)
Diastolic blood pressure, mmHg	74.0 (10.0)	73.0 (9.7)
Total-to-HDL cholesterol ratio	3.8 (1.1)	3.9 (1.3)
Lipid-modifying medication, No (%)	244 (22.8)	28 (21.4)
Antihypertensive medication, No (%)	683 (63.9)	90 (68.7)
Plasma total tau, pg/ml	2.61 (1.97; 3.44)	2.72 (1.83; 2.78)
Plasma NfL, pg/ml	22.1 (16.8; 29.9)	27.7 (18.8; 39.1)
Plasma GFAP, pg/ml	176.8 (130.1; 233.1)	206.7 (157.6; 279.3)
Incident dementia, No (%)	225 (21.0)	3 (3.6)
TBV[a], %	72.2 (3.8)	71.0 (4.6)
SVD burden score [b]	0.7 (0.8)	0.9 (1.0)
WMHV[a], %	0.9 (0.5; 1.7)	1.1 (0.7; 2.0)
Highest quartile of WMHV, No (%)	262 (24.5)	39 (29.8)
Subcortical infarcts, No (%)	131 (12.3)	17 (13.0)
Cerebral microbleeds, No (%)	123 (11.5)	22 (17.9)
Large perivascular spaces, No (%)	191 (17.9)	31 (23.7)
Plasma NfL, pg/ml	22.1 (16.8; 29.9)	27.7 (18.8; 39.1)
Plasma GFAP, pg/ml	176.8 (130.1; 233.1)	206.7 (157.6; 279.3)
Plasma t-tau, pg/ml	2.61 (1.97; 3.44)	2.72 (1.83; 2.78)

Data are means (standard deviation) or median (interquartile range).
Abbreviations: HDL, high-density lipoprotein; TBV, total brain volume; SVD, cerebral small vessel disease; WMHV, white matter hyperintensity volume; NfL, neurofilament light; GFAP, glial fibrillary acidic protein; t-tau, total tau.
[a] TBV and WMHV were expressed as percentage of intracranial volume.
[b] SVD burden score was calculated by assigning one point per cerebral small vessel disease marker based on the following cut-offs (range 0-4): WMHV highest quartile vs lowest three quartiles, and for subcortical infarcts, cerebral microbleeds, and large perivascular spaces presence vs absence.

Supplementary Table 4. Characteristics of the total study population, and according to tertiles of plasma GFAP

Characteristics	Total study population (n=1,069)	Tertiles of plasma GFAP		
		Lowest tertile (n=356, 33.3%)	Middle tertile (n=356, 33.3%)	Highest tertile (n=357, 33.4%)
Age at baseline, years	76.1 (5.4)	73.4 (4.3)	76.2 (5.2)	78.8 (5.4)
Female, No (%)	598 (55.9)	153 (43.0)	198 (55.6)	247 (69.2)
Education level				
- Primary, No (%)	244 (22.8)	70 (19.7)	78 (21.9)	96 (26.9)
- Secondary, No (%)	523 (48.9)	179 (50.3)	179 (50.3)	165 (46.2)
- College/university, No (%)	302 (28.3)	107 (30.1)	99 (27.8)	96 (26.9)
Smoking history				
- Never smoker, No (%)	445 (41.6)	117 (32.9)	154 (43.3)	174 (48.7)
- Former smoker, No (%)	487 (45.6)	169 (47.5)	166 (46.6)	152 (42.6)
- Current smoker, No (%)	137 (12.8)	70 (19.7)	36 (10.1)	31 (8.7)
Type 2 diabetes, No (%)	109 (10.2)	46 (12.9)	33 (9.3)	30 (8.4)
Hypertension, No (%)	866 (81.0)	285 (80.1)	279 (78.4)	302 (84.6)
Stroke				
- Baseline, No (%)	49 (4.6)	15 (4.2)	9 (2.5)	25 (7.0)
- Incident, No (%)	96 (9.0)	28 (7.9)	22 (6.2)	46 (12.9)
Body mass index, kg/m^2	27.0 (4.4)	28.0 (4.5)	27.1 (4.4)	26.1 (4.2)
Systolic blood pressure, mmHg	142.2 (20.1)	141.0 (19.6)	141.3 (20.5)	144.4 (20.1)
Diastolic blood pressure, mmHg	74.0 (10.0)	75.6 (9.7)	74.5 (10.0)	72.0 (9.8)
Total-to-HDL cholesterol ratio	3.8 (1.1)	3.9 (1.1)	3.7 (1.1)	3.6 (1.0)
Lipid-modifying medication, No (%)	244 (22.8)	89 (25.0)	76 (21.3)	79 (22.1)
Antihypertensive medication, No (%)	683 (63.9)	226 (63.5)	211 (59.3)	246 (68.9)
Incident dementia, No (%)	225 (21.0)	31 (8.7)	64 (18.0)	130 (36.4)
TBV[a], %	72.2 (3.8)	72.8 (3.6)	72.3 (4.0)	71.5 (3.6)
SVD burden score [b]	0.7 (0.8)	0.6 (0.8)	0.6 (0.8)	0.8 (0.9)
WMHV[a], %	0.9 (0.5; 1.7)	0.8 (0.4; 1.5)	0.8 (0.4; 1.3)	1.2 (0.6; 2.2)
Highest quartile of WMHV, No (%)	262 (24.5)	74 (20.8)	57 (16.0)	131 (36.7)
Subcortical infarcts, No (%)	131 (12.3)	44 (12.4)	38 (10.7)	49 (13.7)
Cerebral microbleeds, No (%)	123 (11.5)	36 (10.1)	41 (11.5)	46 (12.9)
Large perivascular spaces, No (%)	191 (17.9)	56 (15.7)	69 (19.4)	66 (18.5)
Plasma NfL, pg/ml	22.1 (16.8; 29.9)	17.3 (14.1; 22.1)	21.2 (18.0; 27.4)	29.6 (23.3; 38.6)
Plasma GFAP, pg/ml	176.8 (130.1; 233.1)	114.4 (97.8; 130.1)	176.8 (159.1; 194.9)	294.2 (232.6; 371.9)
Plasma t-tau, pg/ml	2.61 (1.97; 3.44)	2.37 (1.77; 3.16)	2.54 (1.90; 3.25)	3.06; 2.23; 3.98)

Data are means (standard deviation) or median (interquartile range).

Abbreviations: GFAP, glial fibrillary acidic protein; HDL, high-density lipoprotein; TBV, total brain volume; SVD, cerebral small vessel disease; WMHV, white matter hyperintensity volume; NfL, neurofilament light; t-tau, total tau.

[a] TBV and WMHV were expressed as percentage of intracranial volume.

[b] SVD burden score was calculated by assigning one point per cerebral small vessel disease marker based on the following cut-offs (range 0-4): WMHV highest quartile vs lowest three quartiles, and for subcortical infarcts, cerebral microbleeds, and large perivascular spaces presence vs absence.

Supplementary Table 5. Characteristics of the total study population, and according to tertiles of plasma t-tau

Characteristics	Total study population (n=1,069)	Tertiles of plasma total tau		
		Lowest tertile (n=355, 33.2%)	Middle tertile (n=358, 33.5%)	Highest tertile (n=356, 33.3%)
Age at baseline, years	76.1 (5.4)	75.3 (5.3)	76.0 (5.2)	77.0 (5.7)
Female, No (%)	598 (55.9)	144 (40.6)	206 (57.5)	248 (69.7)
Education level				
- Primary, No (%)	244 (22.8)	64 (18.0)	80 (22.3)	100 (28.1)
- Secondary, No (%)	523 (48.9)	173 (48.7)	172 (48.0)	178 (50.0)
- College/university, No (%)	302 (28.3)	118 (33.2)	106 (29.6)	78 (21.9)
Smoking history				
- Never smoker, No (%)	445 (41.6)	136 (38.3)	150 (41.9)	159 (44.7)
- Former smoker, No (%)	487 (45.6)	174 (49.0)	167 (46.6)	146 (41.0)
- Current smoker, No (%)	137 (12.8)	45 (12.7)	41 (11.5)	51 (14.3)
Type 2 diabetes, No (%)	109 (10.2)	40 (11.3)	34 (9.5)	35 (9.8)
Hypertension, No (%)	866 (81.0)	284 (80.0)	286 (80.1)	296 (83.1)
Stroke				
- Baseline, No (%)	49 (4.6)	8 (2.3)	12 (3.4)	29 (8.1)
- Incident, No (%)	96 (9.0)	19 (5.4)	37 (10.3)	40 (11.2)
Body mass index, kg/m^2	27.0 (4.4)	26.7 (4.1)	26.8 (4.3)	27.6 (4.8)
Systolic blood pressure, mmHg	142.2 (20.1)	141.3 (19.0)	142.4 (19.5)	143.0 (21.7)
Diastolic blood pressure, mmHg	74.0 (10.0)	75.8 (10.0)	74.3 (9.6)	71.9 (9.9)
Total-to-HDL cholesterol ratio	3.8 (1.1)	3.7 (1.1)	3.8 (1.1)	3.8 (1.1)
Lipid-modifying medication, No (%)	244 (22.8)	83 (23.4)	69 (19.3)	92 (25.8)
Antihypertensive medication, No (%)	683 (63.9)	221 (62.3)	217 (60.6)	245 (68.8)
Incident dementia, No (%)	225 (21.0)	65 (18.3)	76 (21.2)	84 (23.6)
TBV[a], %	72.2 (3.8)	72.1 (3.9)	72.2 (3.5)	72.2 (3.9)
SVD burden score [b]	0.7 (0.8)	0.6 (0.8)	0.6 (0.8)	0.8 (0.9)
WMHV[a], %	0.9 (0.5; 1.7)	0.8 (0.5; 1.4)	0.8 (0.4; 1.6)	1.1 (0.6; 2.0)
Highest quartile of WMHV, No (%)	262 (24.5)	67 (18.9)	84 (23.5)	111 (31.2)
Subcortical infarcts, No (%)	131 (12.3)	38 (10.7)	38 (10.6)	55 (15.4)
Cerebral microbleeds, No (%)	123 (11.5)	41 (11.5)	39 (10.9)	43 (12.1)
Large perivascular spaces, No (%)	191 (17.9)	62 (17.5)	65 (18.2)	64 (18.0)
Plasma NfL, pg/ml	22.1 (16.8; 29.9)	19.6 (15.4; 26.3)	20.9 (16.4; 27.8)	26.9 (19.1; 34.5)
Plasma GFAP, pg/ml	176.8 (130.1; 233.1)	157.3 (118.6; 208.8)	170.9 (130.7; 221.8)	204.6 (145.1; 299.6)
Plasma t-tau, pg/ml	2.61 (1.97; 3.44)	1.72 (1.39; 1.97)	2.61 (2.39; 2.91)	3.84 (3.44; 4.61)

Data are means (standard deviation) or median (interquartile range).

Abbreviations: t-tau, total tau; HDL, high-density lipoprotein; TBV, total brain volume; SVD, cerebral small vessel disease; WMHV, white matter hyperintensity volume; NfL, neurofilament light; GFAP, glial fibrillary acidic protein.

[a] TBV and WMHV were expressed as percentage of intracranial volume.

[b] SVD burden score was calculated by assigning one point per cerebral small vessel disease marker based on the following cut-offs (range 0-4): WMHV highest quartile vs lowest three quartiles, and for subcortical infarcts, cerebral microbleeds, and large perivascular spaces presence vs absence.

Supplementary Table 6. Association between the SVD burden score[a] on an ordinal scale and incident dementia

SVD burden score[a]	N/events	Hazard ratio (95% confidence interval)
0	567/91	Reference
1	344/78	1.28 (0.94; 1.75)
2	119/40	1.92 (1.30; 2.84)
3	31/12	2.27 (1.22; 4.23)
4	8/4	4.21 (1.50; 11.86)

Adjusted for age, sex, education level, diabetes status, smoking history, body mass index, total cholesterol-to-HDL cholesterol ratio, use of lipid-modifying medication, systolic blood pressure, and use of antihypertensive medication.

Abbreviations: SVD, cerebral small vessel disease.

[a] SVD burden score was calculated by assigning one point per cerebral small vessel disease marker based on the following cut-offs (range 0-4): WMHV highest quartile vs lowest three quartiles, and for subcortical infarcts, cerebral microbleeds, and large perivascular spaces presence vs absence.

Supplementary Table 7. Association between plasma NfL, GFAP, and t-tau and the SVD burden score[a] on an ordinal scale

SVD burden score[a]	N	NfL	GFAP	T-tau
		Odds ratio (95% confidence interval)		
0	567	Reference	Reference	Reference
1	344	1.04 (0.74; 1.46)	1.30 (0.94; 1.79)	1.26 (0.92; 1.73)
2	119	2.27 (1.47; 3.49)	1.47 (0.93; 2.34)	1.61 (1.03; 2.50)
3	31	3.47 (1.90; 6.37)	1.07 (0.45; 2.53)	1.22 (0.55; 2.72)
4	8	4.39 (1.65; 11.67)	1.31 (0.26; 6.51)	1.55 (0.40; 6.06)

Odds ratios are expressed per natural log-transformed pg/ml higher plasma NfL, GFAP or t-tau. Adjusted for age, sex, education level, diabetes status, smoking history, body mass index, total cholesterol-to-HDL cholesterol ratio, use of lipid-modifying medication, systolic blood pressure, and use of antihypertensive medication.
Abbreviations: SVD, cerebral small vessel disease.
[a] SVD burden score was calculated by assigning one point per cerebral small vessel disease marker based on the following cut-offs (range 0-4): WMHV highest quartile vs lowest three quartiles, and for subcortical infarcts, cerebral microbleeds, and large perivascular spaces presence vs absence.

Supplementary Table 8. Total effects, direct effects, and explained effects by WMHV[a], subcortical infarcts, cerebral microbleeds, and large perivascular spaces of the associations between plasma NfL, GFAP, and t-tau and incident dementia

Plasma biomarker	SVD marker	Total effects	Direct effects	Explained effects
		Hazard ratio (95% confidence interval)		
NfL	WMHV[a]	2.04 (1.55; 2.68)	1.92 (1.44; 2.54)	1.32 (1.06; 1.85)
	Subcortical infarcts	2.04 (1.55; 2.68)	1,95 (1.47; 2.59)	1.30 (0.98; 1.99)
	Cerebral microbleeds	2.04 (1.55; 2.68)	2.01 (1.52; 2.66)	1.13 (0.96; 1.49)
	Perivascular spaces	2.04 (1.55; 2.68)	2.02 (1.54; 2.66)	1.02 (0.93; 1.16)
GFAP	WMHV[a]	2.79 (2.16; 3.61)	2.69 (2.08; 3.49)	1.17 (1.01; 1.48)
	Subcortical infarcts	2.79 (2.16; 3.61)	2.83 (2.18; 3.68)	1.04 (0.84; 1.38)
	Cerebral microbleeds	2.79 (2.16; 3.61)	2.73 (2.11; 3.53)	1.04 (0.87; 1.25)
	Perivascular spaces	2.79 (2.16; 3.61)	2.78 (2.16; 3.59)	0.99 (0.87; 1.12)
T-tau	WMHV[a]	1.16 (0.85; 1.59)	1.13 (0.83; 1.55)	1.18 (1.01; 1.42)
	Subcortical infarcts	1.16 (0.85; 1.59)	1.12 (0.81; 1.53)	1.13 (0.93; 1.56)
	Cerebral microbleeds	1.16 (0.85; 1.59)	1.17 (0.85; 1.60)	1.04 (0.89; 1.31)
	Perivascular spaces	1.16 (0.85; 1.59)	1.17 (0.86; 1.60)	1.02 (0.91; 1.15)

Hazard ratios for incident dementia are expressed per natural log-transformed pg/ml higher plasma NfL, GFAP or t-tau. Total effect, direct effect and explained effect are defined in Supplementary Figure 2. The explained effect quantifies the degree to which the SVD markers attenuated the association of plasma biomarkers with incident dementia. All analyses adjusted for age, sex, education level, diabetes status, smoking history, body mass index, total cholesterol-to-HDL cholesterol ratio, use of lipid-modifying medication, systolic blood pressure, and use of antihypertensive medication.

Abbreviations: SVD, cerebral small vessel disease; NfL, neurofilament light; GFAP, glial fibrillary acidic protein; t-tau, total tau; WMHV, white matter hyperintensity volume.

[a] Highest quartile versus lowest three quartiles of WMHV.

Supplementary Table 9. Total effects, direct effects, and explained effects by the SVD burden score[a] of the associations between plasma NfL, GFAP, and t-tau and incident dementia – additional adjustment for TBV[b] and prevalent or incident stroke

Plasma biomarker	Total effects		Direct effects		Explained effects	
	Hazard ratio (95% confidence interval)					
	Additional adjustment for TBV[b]	Additional adjustment for prevalent and incident stroke	Additional adjustment for TBV[b]	Additional adjustment for prevalent and incident stroke	Additional adjustment for TBV[b]	Additional adjustment for prevalent and incident stroke
NfL	1.91 (1.46; 2.51)	1.92 (1.44; 2.55)	1.74 (1.31; 2.31)	1.76 (1.31; 2.37)	1.07 (1.02; 1.15)	1.06 (1.01; 1.12)
GFAP	2.72 (2.11; 3.51)	2.86 (2.20; 3.71)	2.62 (2.04; 3.36)	2.76 (2.20; 3.71)	1.03 (0.99; 1.07)	1.02 (0.99; 1.06)
T-tau	1.13 (0.83; 1.54)	1.08 (2.20; 3.71)	1.10 (0.81; 1.50)	1.07 (0.78; 1.47)	1.04 (0.99; 1.08)	1.02 (0.99; 1.07)

Hazard ratios for incident dementia are expressed per natural log-transformed pg/ml higher plasma NfL, GFAP or t-tau. Total effect, direct effect and explained effect are defined in Supplementary Figure 2. The explained effect quantifies the degree to which the SVD burden score[a] attenuated the association of plasma biomarkers with incident dementia. All analyses adjusted for age, sex, education level, diabetes status, smoking history, body mass index, total cholesterol-to-HDL cholesterol ratio, use of lipid-modifying medication, systolic blood pressure, and use of antihypertensive medication.

Abbreviations: SVD, cerebral small vessel disease; TBV, total brain volume; NfL, neurofilament light; GFAP, glial fibrillary acidic protein; t-tau, total tau;

[a] SVD burden score was calculated by assigning one point per cerebral small vessel disease marker based on the following cut-offs (range 0-4): WMHV highest quartile vs lowest three quartiles, and for subcortical infarcts, cerebral microbleeds, and large perivascular spaces presence vs absence.

[b] TBV was expressed as percentage of intracranial volume.

Supplementary Table 10. Total effects, direct effects, and explained effects by WMHV[b], subcortical infarcts, cerebral microbleeds, and large perivascular spaces of the associations between plasma NfL, GFAP, and t-tau and incident dementia – additional adjustment for TBV[a] and prevalent or incident stroke

Plasma biomarker	SVD marker	Total effects		Indirect effects		Explained effects	
		Hazard ratio (95% confidence interval)					
		Additional adjustment for TBV[a]	Additional adjustment for prevalent and incident stroke	Additional adjustment for TBV[a]	Additional adjustment for prevalent and incident stroke	Additional adjustment for TBV[a]	Additional adjustment for prevalent and incident stroke
NfL	WMHV[b]	1.91 (1.46; 2.51)	1.92 (1.44; 2.55)	1.77 (1.34; 2.34)	1.82 (1.36; 2.43)	1.31 (1.10; 2.03)	1.25 (1.02; 1.69)
	Subcortical infarcts	1.91 (1.46; 2.51)	1.92 (1.44; 2.55)	1.84 (1.39; 2.43)	1.85 (1.38; 2.48)	1.28 (0.79; 1.78)	1.20 (0.93; 1.73)
	Cerebral microbleeds	1.91 (1.46; 2.51)	1.92 (1.44; 2.55)	1.90 (1.44; 2.49)	1.88 (1.41; 2.51)	1.11 (0.93; 1.47)	1.12 (0.95; 1.44)
	Perivascular spaces	1.91 (1.46; 2.51)	1.92 (1.44; 2.55)	1.90 (1.45; 2.49)	1.90 (1.43; 2.53)	1.02 (0.94; 1.12)	1.03 (0.94; 1.16)
GFAP	WMHV[b]	2.72 (2.11; 3.51)	2.86 (2.20; 3.71)	2.61 (2.03; 3.37)	2.77 (2.13; 3.60)	1.21 (1.02; 1.57)	1.14 (0.99; 1.41)
	Subcortical infarcts	2.72 (2.11; 3.51)	2.86 (2.20; 3.71)	2.74 (2.12; 3.55)	2.89 (2.22; 3.76)	1.03 (0.84; 1.33)	1.01 (0.83; 1.28)
	Cerebral microbleeds	2.72 (2.11; 3.51)	2.86 (2.20; 3.71)	2.68 (2.08; 3.46)	2.80 (2.15; 3.63)	1.03 (0.87; 1.23)	1.04 (0.86; 1.25)
	Perivascular spaces	2.72 (2.11; 3.51)	2.86 (2.20; 3.71)	2.72 (2.11; 3.49)	2.84 (2.20; 3.69)	0.99 (0.88; 1.10)	1.00 (0.87; 1.12)
T-tau	WMHV[b]	1.13 (0.83; 1.54)	1.08 (0.79; 1.49)	1.10 (0.80; 1.49)	1.05 (0.76; 1.45)	1.21 (1.01; 1.57)	1.12 (0.96; 1.37)
	Subcortical infarcts	1.13 (0.83; 1.54)	1.08 (0.79; 1.49)	1.09 (0.80; 1.49)	1.05 (0.77; 1.45)	1.12 (0.93; 1.54)	1.07 (0.88; 1.36)
	Cerebral microbleeds	1.13 (0.83; 1.54)	1.08 (0.79; 1.49)	1.12 (0.82; 1.53)	1.09 (0.79; 1.50)	1.03 (0.90; 1.28)	1.03 (0.87; 1.27)
	Perivascular spaces	1.13 (0.83; 1.54)	1.08 (0.79; 1.49)	1.13 (0.83; 1.54)	1.10 (0.80; 1.50)	1.01 (0.92; 1.13)	1.02 (0.91; 1.16)

Hazard ratios for incident dementia are expressed per natural log-transformed pg/ml higher plasma NfL, GFAP or t-tau. Total effect, direct effect and explained effect are defined in Supplementary Figure 2. The explained effect quantifies the degree to which the SVD markers attenuated the association of plasma biomarkers with incident dementia. All analyses adjusted for age, sex, education level, diabetes status, smoking history, body mass index, total cholesterol-to-HDL cholesterol ratio, use of lipid-modifying medication, systolic blood pressure, and use of antihypertensive medication.
Abbreviations: SVD, cerebral small vessel disease; WMHV, white matter hyperintensity volume; NfL, neurofilament light; GFAP, glial fibrillary acidic protein; t-tau, total tau; TBV, total brain volume.
[a] TBV was expressed as percentage of intracranial volume.
[b] Highest quartile versus lowest three quartiles of WMHV.

Supplementary Table 11. Total effects, direct effects, and explained effects by WMHV[a] on a continuous scale and expressed as higher versus lower than the median of the associations between plasma NfL, GFAP, and t-tau and incident dementia

Plasma biomarker	WMHV	Total effect	Direct effect	Explained effects
		Hazard ratio (95% confidence interval)		
NfL	WMHV[a] on a continuous scale	2.04 (1.55; 2.68)	1.84 (1.38; 2.45)	1.09 (1.04; 1.18)
	WMHV expressed as higher versus lower than the median	2.04 (1.55; 2.68)	1.84 (1.38; 2.45)	1.09 (1.04; 1.18)
GFAP	WMHV[a] on a continuous scale	2.79 (2.16; 3.61)	2.71 (2.10; 3.61)	1.05 (1.01; 1.12)
	WMHV expressed as higher versus lower than the median	2.79 (2.16; 3.61)	2.77 (2.13; 3.59)	1.13 (0.96; 1.40)
T-tau	WMHV[a] on a continuous scale	1.16 (0.85; 1.59)	1.15 (0.85; 1.59)	1.04 (1.00; 1.09)
	WMHV expressed as higher versus lower than the median	1.16 (0.85; 1.59)	1.13 (0.83; 1.55)	1.16 (1.00; 1.45)

Hazard ratios for incident dementia are expressed per natural log-transformed pg/ml higher plasma NfL, GFAP or t-tau. Total effect, direct effect and explained effect are defined in Supplementary Figure 2. The explained effect quantifies the degree to which WMHV explained the association of plasma biomarkers with incident dementia. All analyses adjusted for age, sex, education level, diabetes status, smoking history, body mass index, total cholesterol-to-HDL cholesterol ratio, use of lipid-modifying medication, systolic blood pressure, and use of antihypertensive medication.
Abbreviations: WMHV white matter hyperintensity volume; NfL, neurofilament light; GFAP, glial fibrillary acidic protein; t-tau, total tau.
[a] WMHV expressed per SD percentage of total intracranial volume.

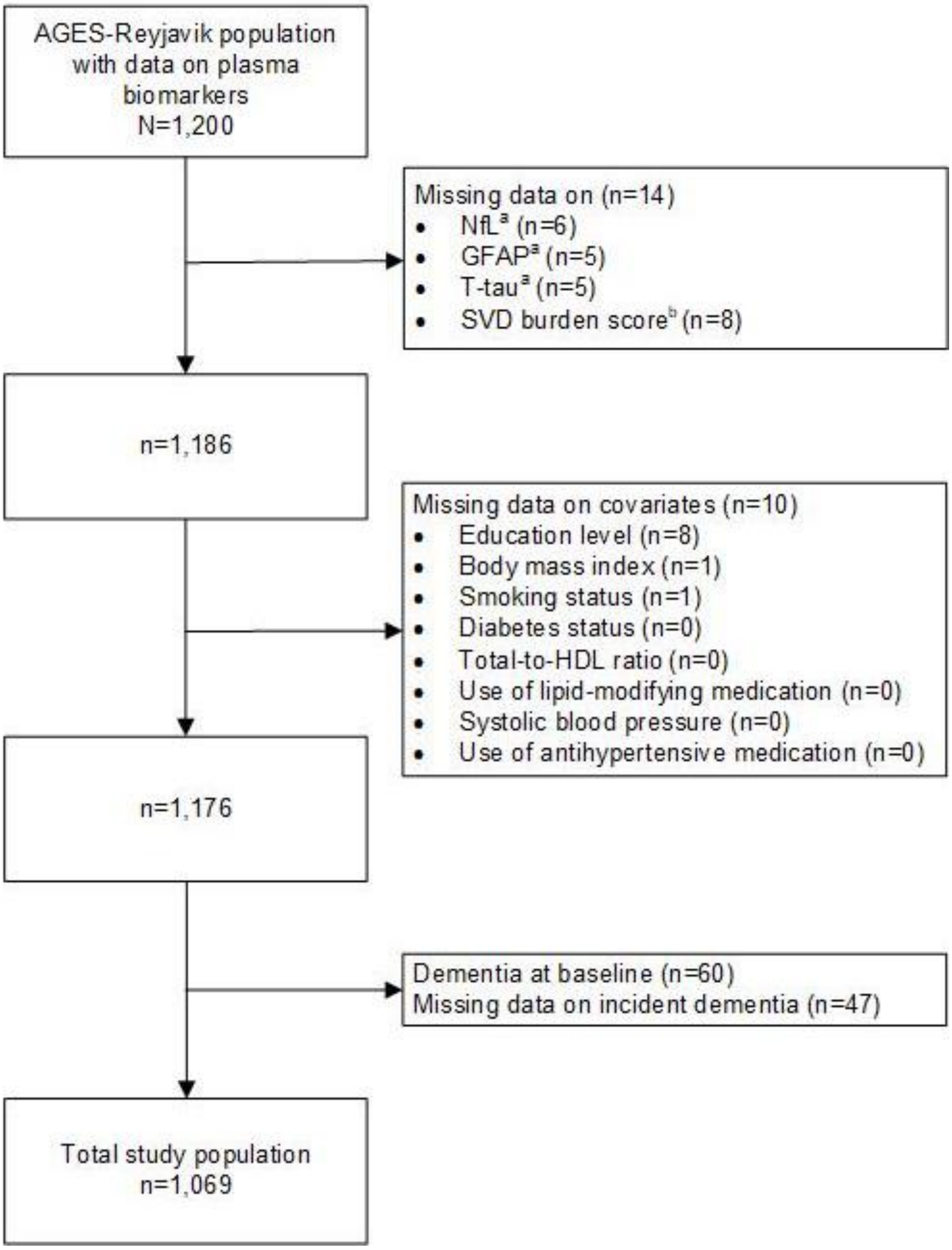

Supplementary Figure 1. Flow chart derivation of the study population
Missing data not mutually exclusive.
Abbreviations: NfL, neurofilament light; GFAP, glial fibrillary acidic protein; t-tau; total tau; TBV, total brain volume; SVD, cerebral small vessel disease; HDL, high-density lipoprotein.
[a] Missing data on plasma biomarkers was due to technical reasons (missing sample (n=1), insufficient volume available (n=3) and invalid result (n=2 for NfL and n=1 for GFAP and t-tau, respectively)).
[b] SVD burden score was calculated by assigning one point per cerebral small vessel disease marker based on the following cut-offs (range 0-4): WMHV highest quartile vs lowest three quartiles, and for subcortical infarcts, cerebral microbleeds, and large perivascular spaces presence vs absence. Missing data on the SVD burden score was due to technical reasons (n=8 did not have specific MRI images needed for assessment of cerebral microbleeds).

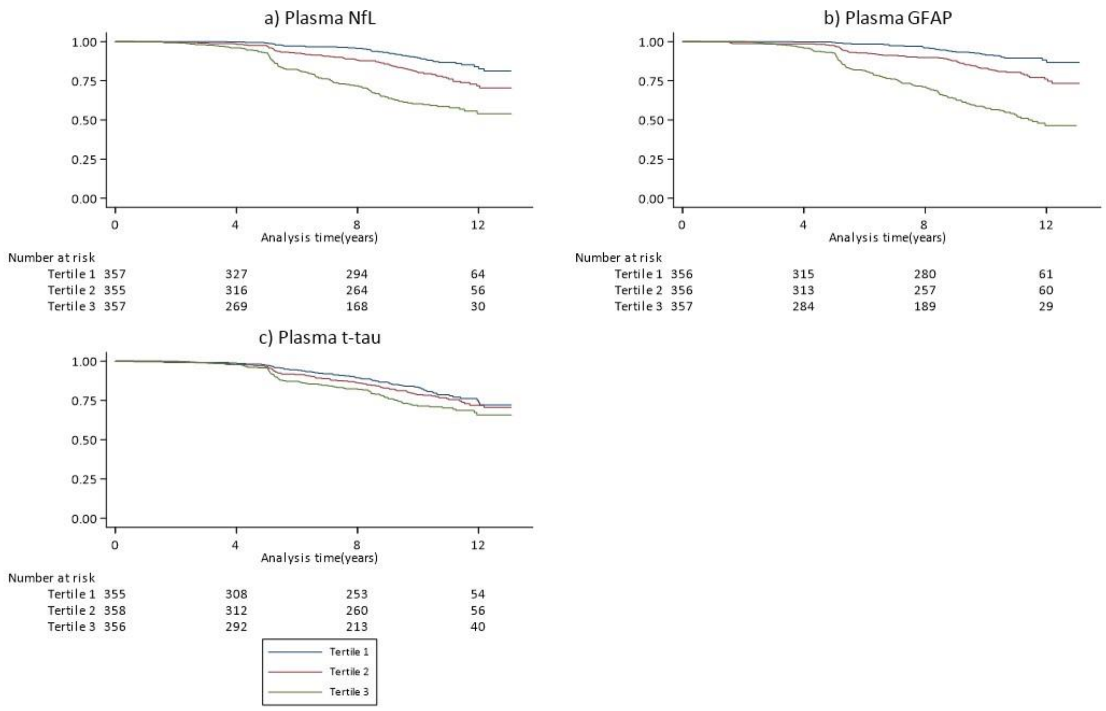

Supplementary Figure 2. Survival curves for incident dementia according to tertiles of plasma NfL (Panel a), GFAP (Panel b), and t-tau (Panel c).
Abbreviations: NfL, neurofilament light; GFAP, glial fibrillary acidic protein; t-tau, total tau.

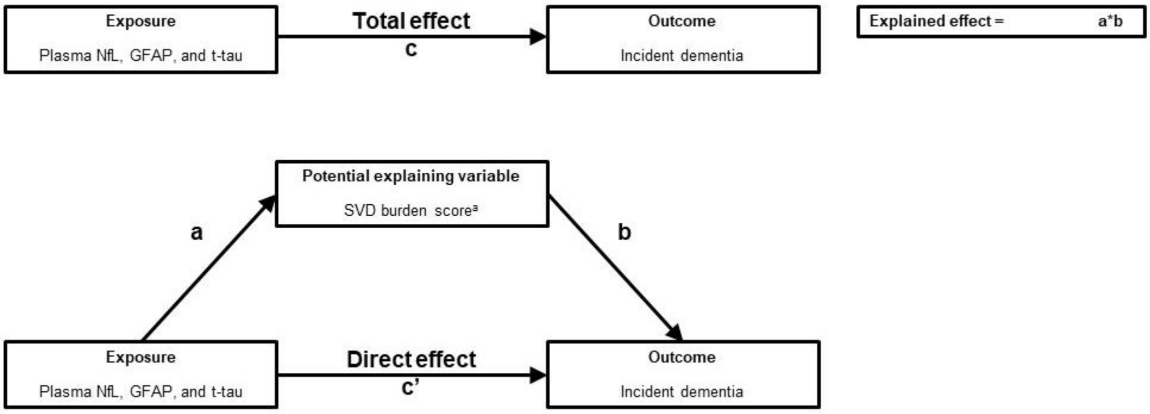

Supplementary Figure 3. Summary of the statistical analysis

The explained effects were calculated as the multiplied effects of plasma biomarkers and the SVD burden score (a) and the SVD burden score and incident dementia, adjusted for the plasma biomarkers (b). In analysis with a dichotomous outcome variable (i.e. incident dementia), the direct effect (c’) will be systematic overestimated due to change of scales of the coefficients in logistic or Cox models [1]. Therefore, calculating the explained effect using a*b is recommended [1].

Abbreviations: NfL, neurofilament light; GFAP, glial fibrillary acidic protein; t-tau, total tau; SVD, cerebral small vessel disease.

[a] SVD burden score was calculated by assigning one point per cerebral small vessel disease marker based on the following cut-offs (range 0-4): WMHV highest quartile vs lowest three quartiles, and for subcortical infarcts, cerebral microbleeds, and large perivascular spaces presence vs absence.

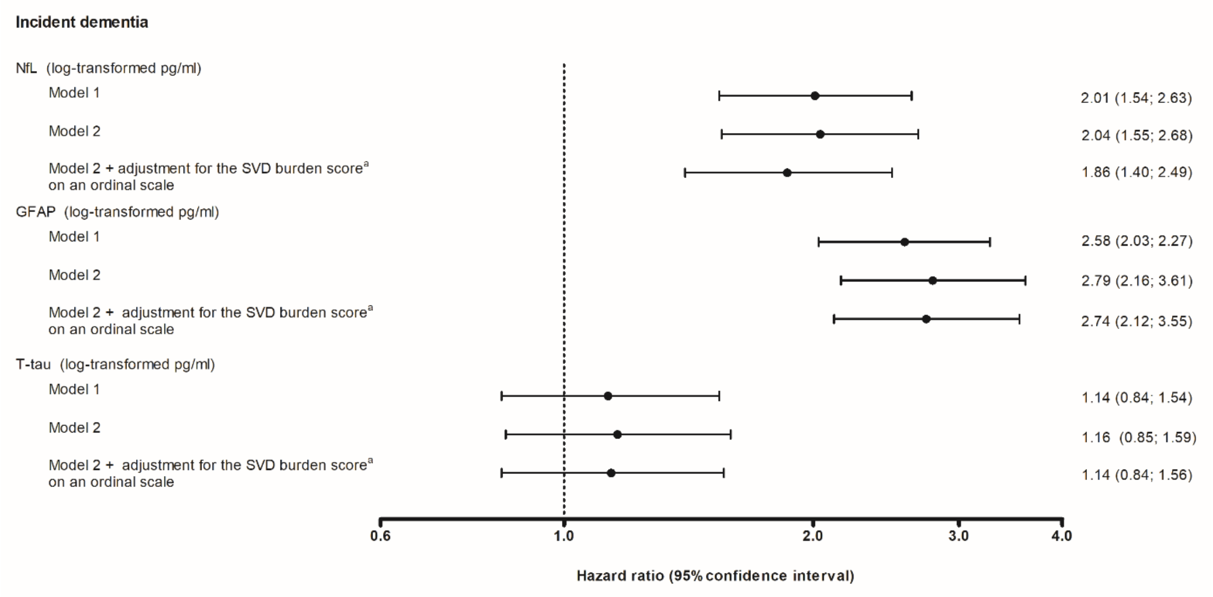

Supplementary Figure 4. Associations between plasma NfL, GFAP, and t-tau and incident dementia with and without adjustment for the SVD burden score[a] on an ordinal scale

Hazard ratios for incident dementia are expressed per natural log-transformed pg/ml higher plasma NfL, GFAP or t-tau. Model 1 adjusted for age and sex. Model 2 additionally adjusted for education level, diabetes status, smoking history, body mass index, total cholesterol-to-HDL cholesterol ratio, use of lipid-modifying medication, systolic blood pressure, and use of antihypertensive medication. Models 1 and 2 represent the total effect, and model 2 + adjustment for the SVD burden score represents the direct effect. Total and direct effect are defined in Supplementary Figure 2.

Abbreviations: NfL, neurofilament light; GFAP, glial fibrillary acidic protein; t-tau, total-tau; SVD, cerebral small vessel disease.

[a] SVD burden score was calculated by assigning one point per cerebral small vessel disease marker based on the following cut-offs (range 0-4): WMHV highest quartile vs lowest three quartiles, and for subcortical infarcts, cerebral microbleeds, and large perivascular spaces presence vs absence.

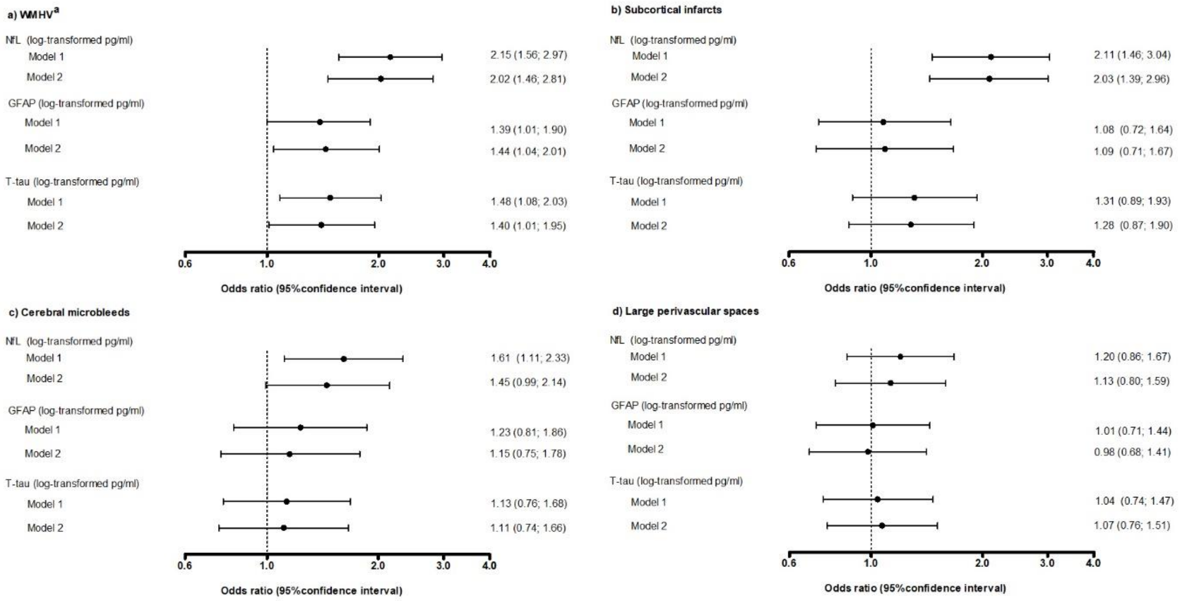

Supplementary Figure 5. Associations between on the one hand plasma NfL, GFAP, and t-tau and on the other WMHV[a] (Panel a), subcortical infarcts (Panel b), cerebral microbleeds (Panel c) and large perivascular spaces (Panel d)

Results are expressed per natural log-transformed pg/ml higher plasma NfL, GFAP or t-tau. Model 1 adjusted for age and sex. Model 2 additionally adjusted for education level, diabetes status, smoking history, body mass index, total cholesterol-to-HDL cholesterol ratio, use of lipid-modifying medication, systolic blood pressure, and use of antihypertensive medication.

Abbreviations: WMHV, white matter hyperintensity volume; NfL, neurofilament light; GFAP, glial fibrillary acidic protein; t-tau, total tau.

[a] Highest quartile versus lowest three quartiles of WMHV.

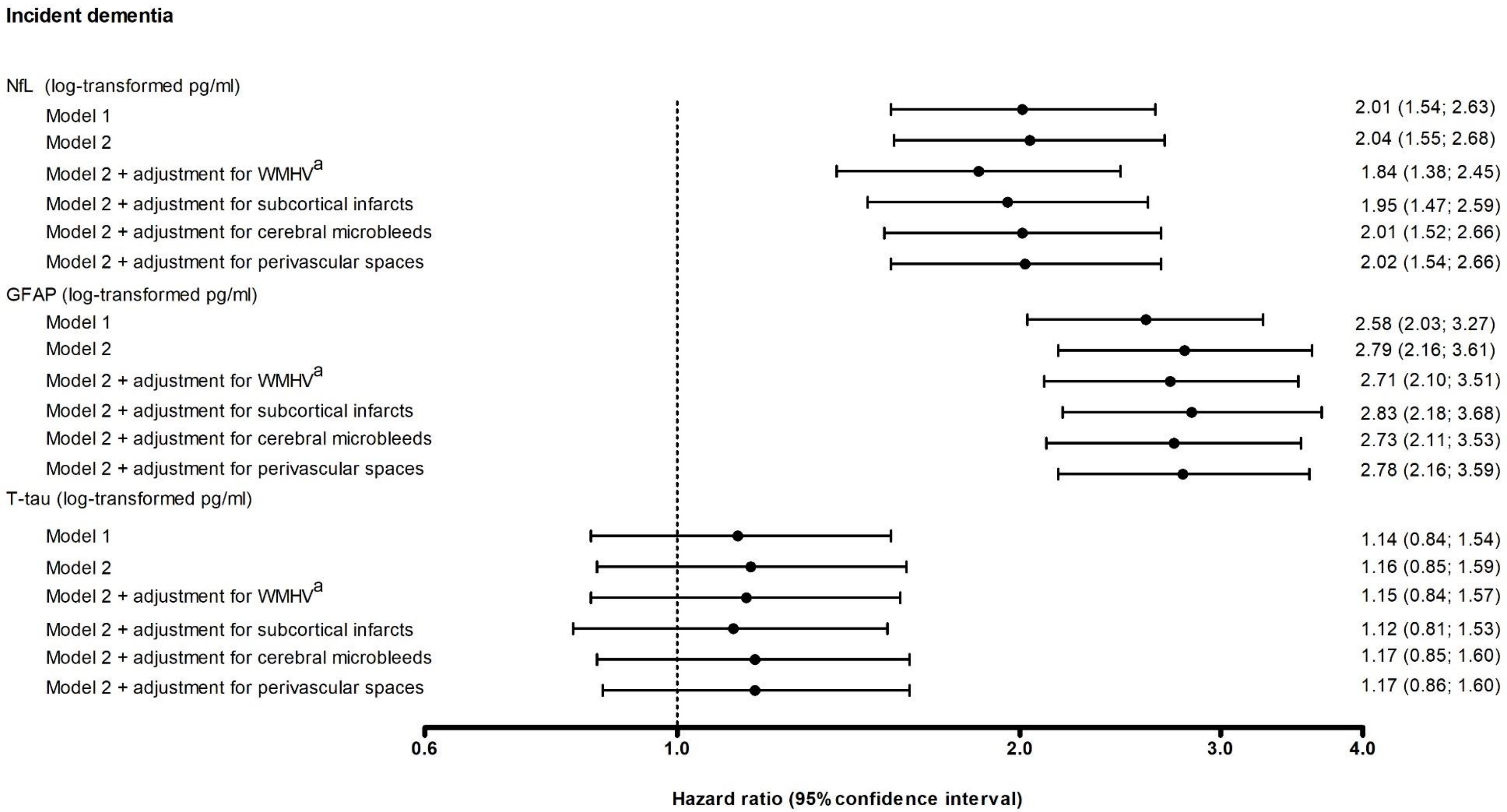

Supplementary Figure 6. Associations between plasma NfL, GFAP, and t-tau and incident dementia with and without adjustment for white matter hyperintensity volume[a], subcortical infarcts, cerebral microbleeds, and large perivascular spaces

Hazard ratios for incident dementia are expressed per natural log-transformed pg/ml higher plasma NfL, GFAP or t-tau. Model 1 adjusted for age and sex. Model 1 adjusted for age and sex. Model 2 additionally adjusted for education level, diabetes status, smoking history, body mass index, total cholesterol-to-HDL cholesterol ratio, use of lipid-modifying medication, systolic blood pressure, and use of antihypertensive medication. Models 1 and 2 represent the total effect, and model 2 + adjustment for the individual SVD markers represents the direct effect. Total and direct effect are defined in Supplementary Figure 2.

Abbreviations: WMHV, white matter hyperintensity volume; ICV, intracranial volume; SD, standard deviation; NfL, neurofilament light; GFAP, glial fibrillary acidic protein; WMHV, white matter hyperintensity.

[a] Highest quartile versus lowest three quartiles of WMHV.

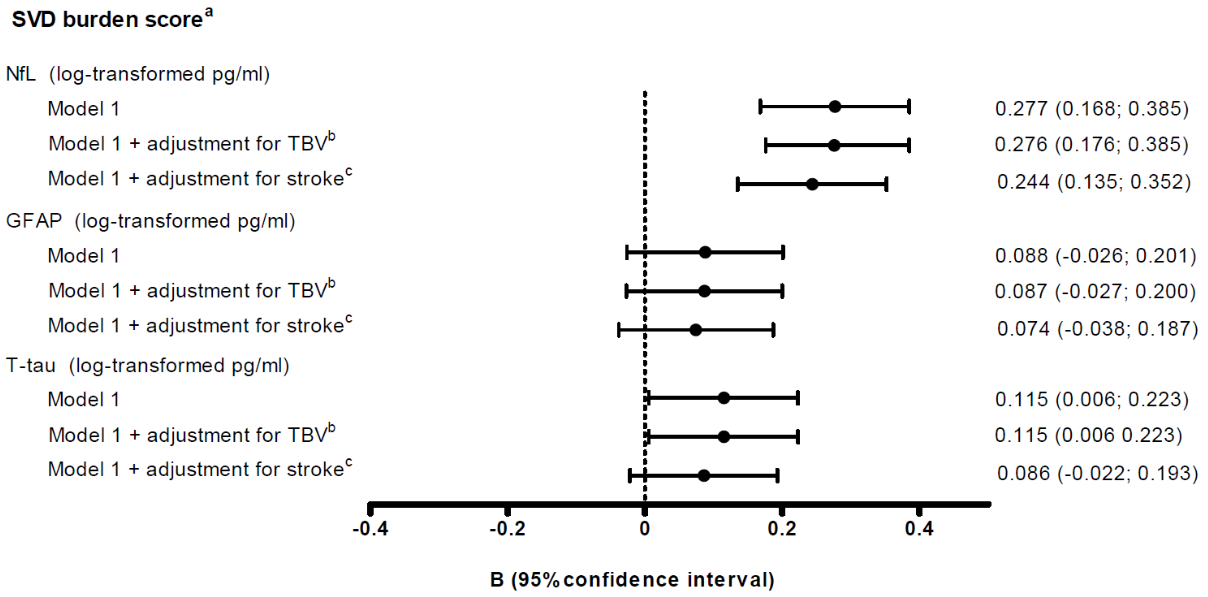

Supplementary Figure 7. Associations between plasma NfL, GFAP, and t-tau and the SVD burden score[a] - additional adjustment for TBV[b] and prevalent or incident stroke

Hazard ratios for incident dementia are expressed per natural log-transformed pg/ml higher plasma NfL, GFAP or t-tau. Model 1 adjusted for age, sex, education level, diabetes status, smoking history, body mass index, total cholesterol-to-HDL cholesterol ratio, use of lipid-modifying medication, systolic blood pressure, and use of antihypertensive medication.

Abbreviations: SVD, cerebral small vessel disease; SD, standard deviation; NfL, neurofilament light; GFAP, glial fibrillary acidic protein; t-tau, total-tau.

[a] SVD burden score was calculated by assigning one point per cerebral small vessel disease marker based on the following cut-offs (range 0-4): WMHV highest quartile vs lowest three quartiles, and for subcortical infarcts, cerebral microbleeds, and large perivascular spaces presence vs absence.

[b] TBV was expressed as percentage of intracranial volume.

‡ Prevalent and incident stroke.

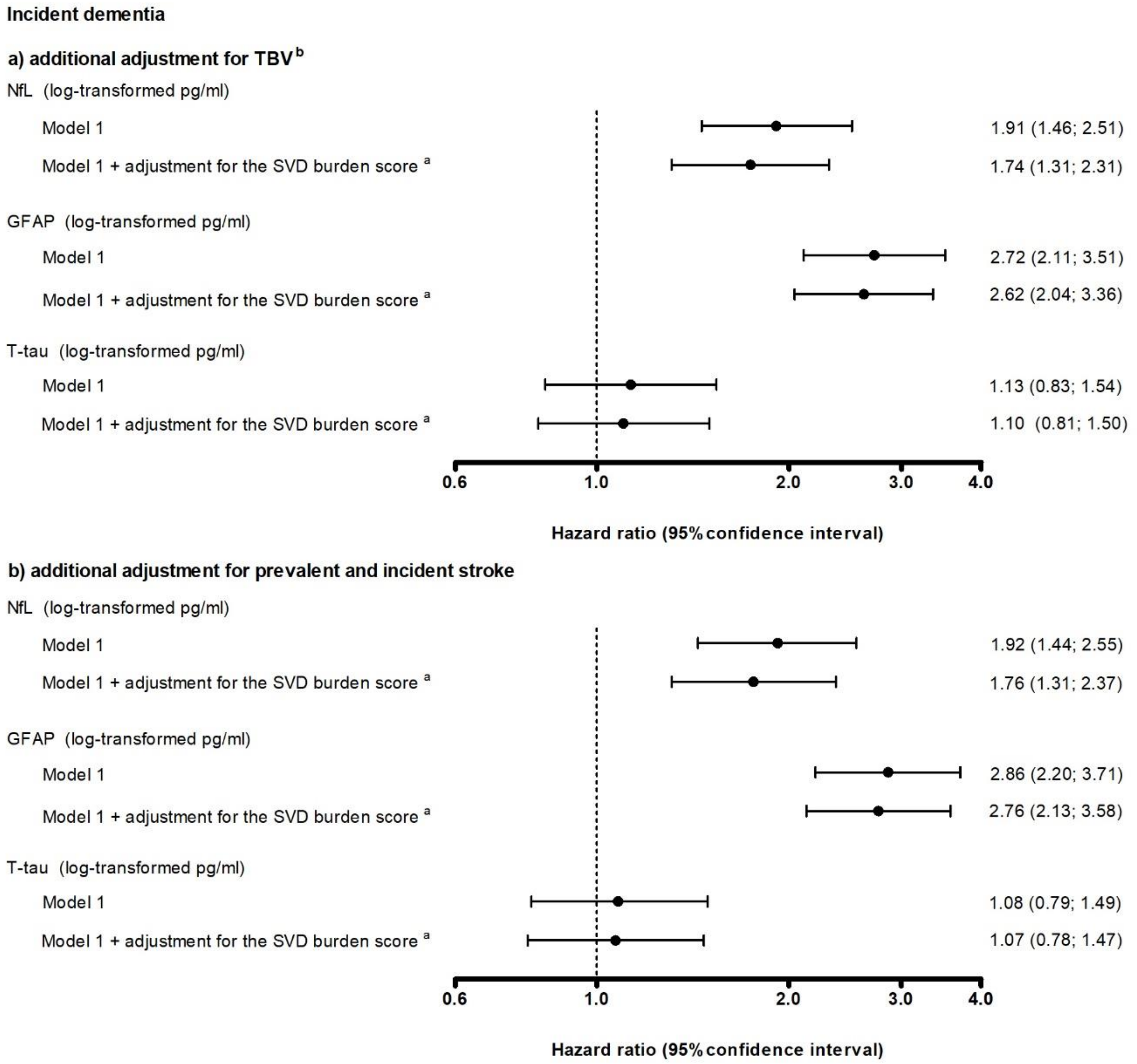

Supplementary Figure 8. Associations between plasma NfL, GFAP, and t-tau and incident dementia with and without adjustment for the SVD burden score[a] - additional adjustment for TBV[b] (Panel a) and prevalent and incident stroke (Panel b)

Hazard ratios for incident dementia are expressed per natural log-transformed pg/ml higher plasma NfL, GFAP or t-tau. Model 1 adjusted for age, education level, diabetes status, smoking history, body mass index, total cholesterol-to-HDL cholesterol ratio, use of lipid-modifying medication, systolic blood pressure, and use of antihypertensive medication. Models 1 represents the total effect, and model 1 + adjustment for the SVD burden score represents the direct effect. Total and direct effect are defined in Supplementary Figure 2.

Abbreviations: TBV, total brain volume; NfL, neurofilament light; GFAP, glial fibrillary acidic protein; t-tau, total tau; SVD, cerebral small vessel disease.

[a] SVD burden score was calculated by assigning one point per cerebral small vessel disease marker based on the following cut-offs (range 0-4): WMHV highest quartile vs lowest three quartiles, and for subcortical infarcts, cerebral microbleeds, and large perivascular spaces presence vs absence.

[b] TBV was expressed as percentage of intracranial volume.

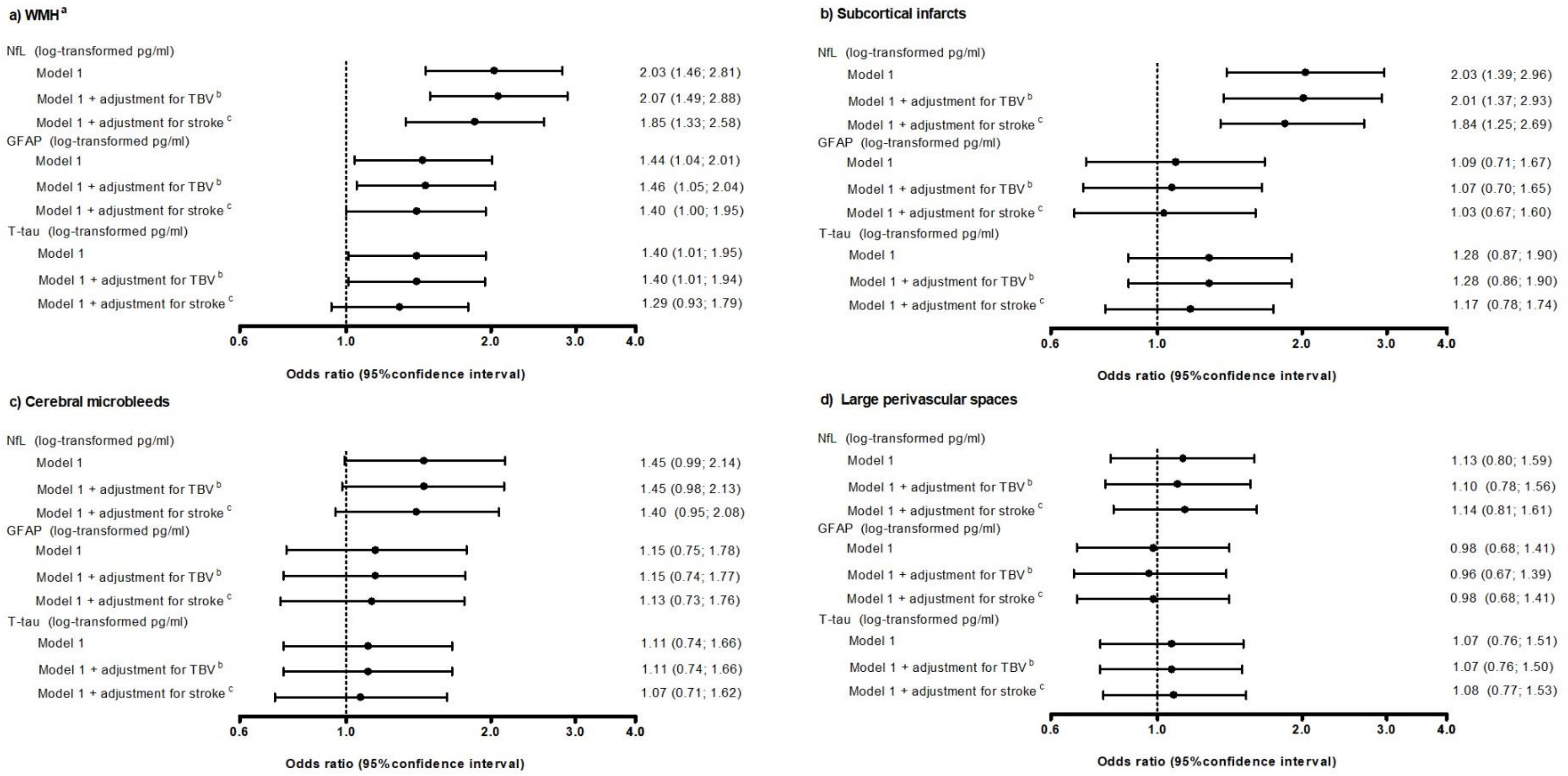

Supplementary Figure 9. Associations between on the one hand plasma NfL, GFAP, and t-tau and on the other WMHV (Panel a), subcortical infarcts (Panel b), cerebral microbleeds (Panel c) and large perivascular spaces (Panel d) - additional adjustment for TBV[b] and prevalent or incident stroke
Results are expressed per natural log-transformed pg/ml higher plasma NfL, GFAP or t-tau. Model 1 adjusted for age and sex, education level, diabetes status, smoking history, body mass index, total cholesterol-to-HDL cholesterol ratio, use of lipid-modifying medication, systolic blood pressure, and use of antihypertensive medication.
Abbreviations: WMHV, white matter hyperintensity volumeNfL, neurofilament light; GFAP, glial fibrillary acidic protein; t-tau, total.

[a] Highest quartile versus lowest three quartiles of WMHV.

[b] TBV was expressed as percentage of intracranial volume.

[c] Prevalent and incident stroke.

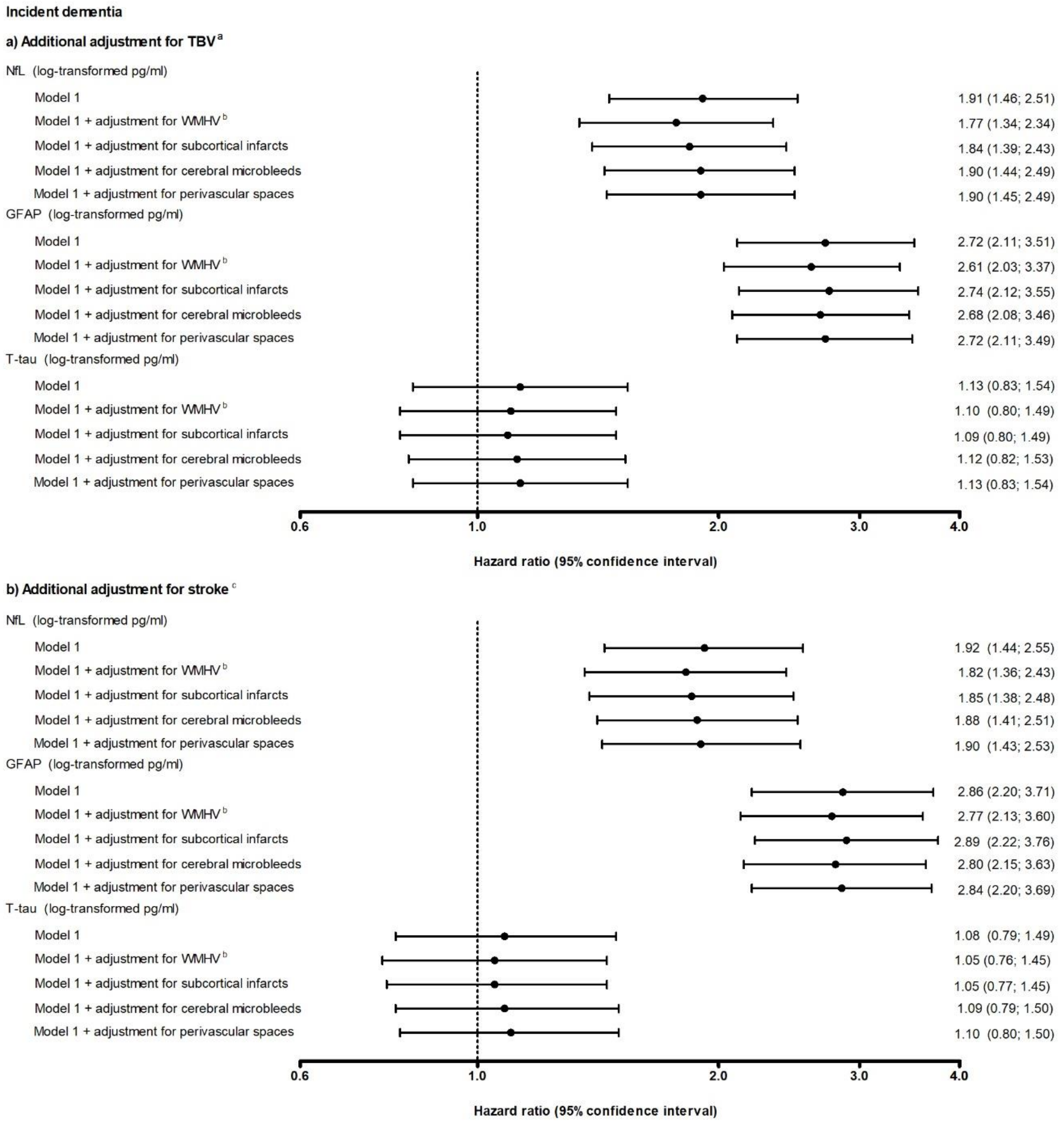

Supplementary Figure 10. Associations between plasma NfL, GFAP, and t-tau and incident dementia with and without adjustment for WMHV[b], subcortical infarcts, cerebral microbleeds, and large perivascular spaces - additional adjustment for TBV[a] (Panel a) and prevalent and incident stroke (Panel b).
Hazard ratios for incident dementia are expressed per natural log-transformed pg/ml higher plasma NfL, GFAP or t-tau. Model 1 adjusted for age and sex, education level, diabetes status, smoking history, body mass index, total cholesterol-to-HDL cholesterol ratio, use of lipid-modifying medication, systolic blood pressure, and use of antihypertensive medication. Models 1 represents the total effect, and model 2 + adjustment for the individual SVD markers represents the direct effect. Total and direct effect are defined in Supplementary Figure 2.
Abbreviations: TBV, total brain volume; WMHV, white matter hyperintensity volume; NfL, neurofilament light; GFAP, glial fibrillary acidic protein; t-tau, total.

[a] TBV was expressed as percentage of intracranial volume.

[b] Highest quartile versus lowest three quartiles of WMHV.

[c] Prevalent and incident stroke.

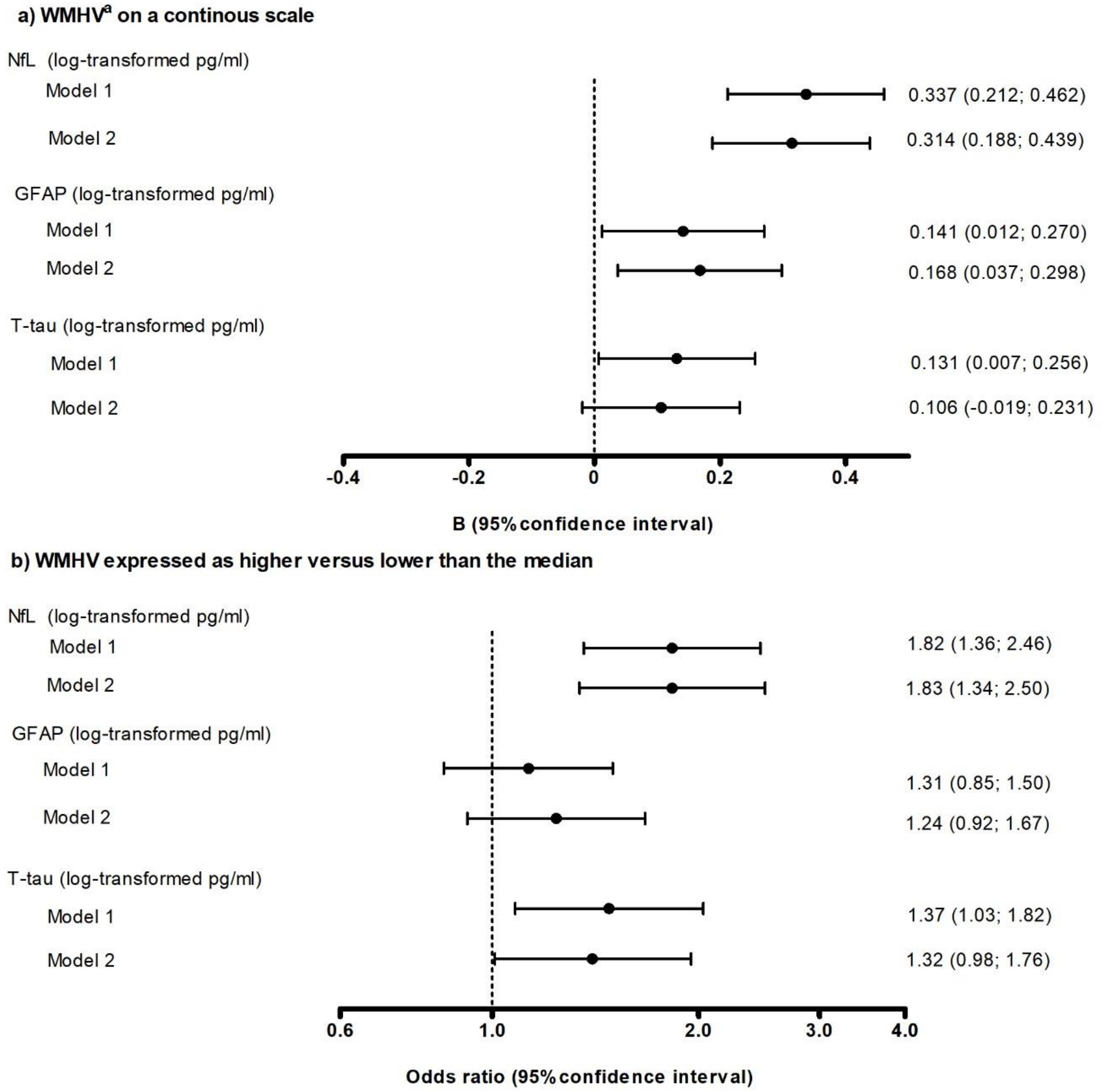

Supplementary Figure 11. Associations between on the one hand plasma NfL, GFAP, and t-tau and on the other WMHV[a] on a continuous scale (Panel a), and expressed as higher versus lower than the median (Panel b)

Betas are expressed per natural log-transformed pg/ml higher plasma NfL, GFAP or t-tau. Model 1 adjusted for age and sex. Model 2 additionally adjusted for education level, diabetes status, smoking history, body mass index, total cholesterol-to-HDL cholesterol ratio, use of lipid-modifying medication, systolic blood pressure, and use of antihypertensive medication.

Abbreviations: WMHV white matter hyperintensity volume; NfL, neurofilament light; GFAP, glial fibrillary acidic protein; t-tau, total-tau.

[a] WMHV expressed per SD percentage of total intracranial volume.

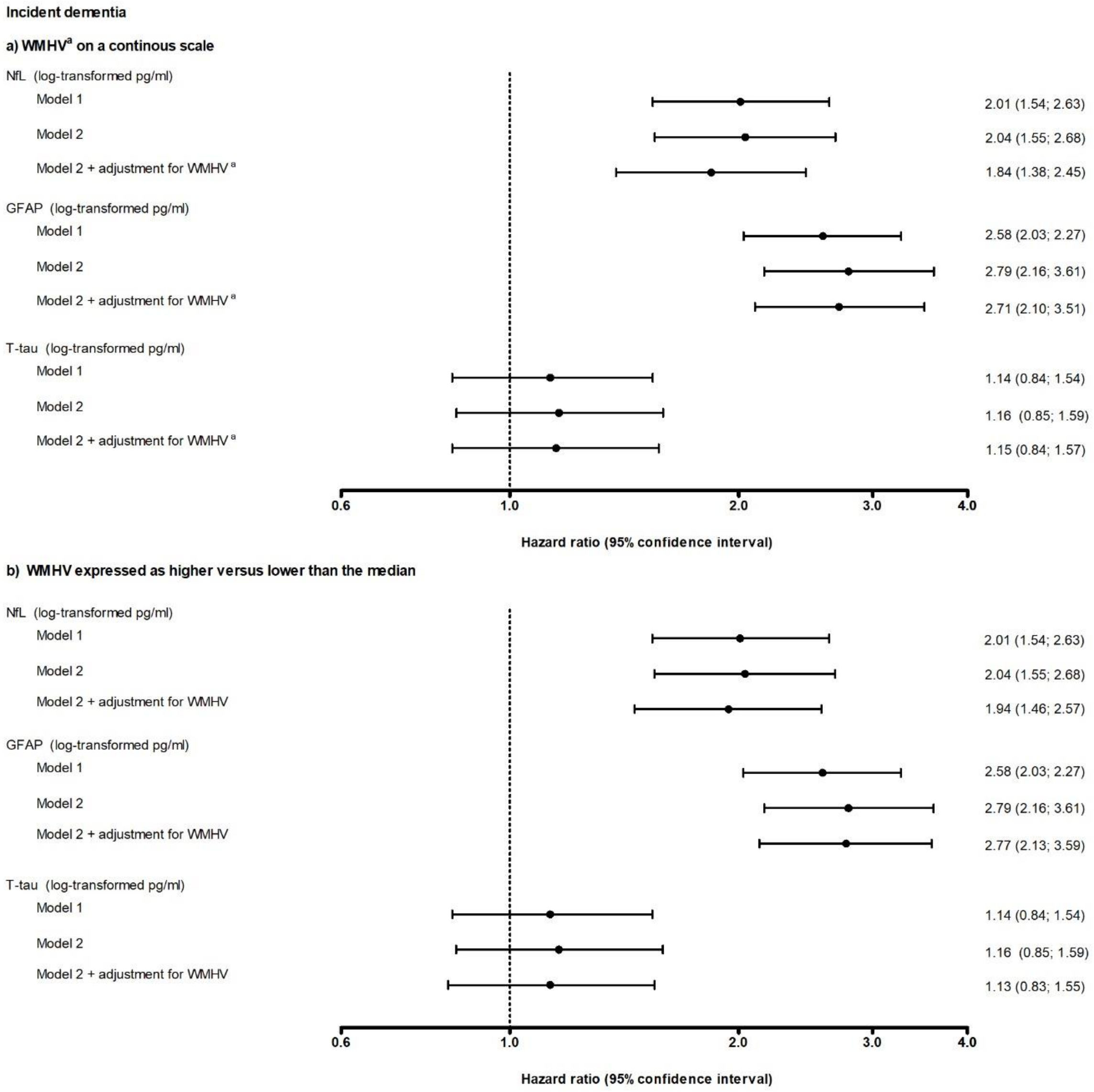

Supplementary Figure 12. Associations between plasma NfL, GFAP, and t-tau and incident dementia with and without adjustment for WMHV[a] on a continuous scale (Panel a), and expressed as higher versus lower than the median (Panel b)

Hazard ratios for incident dementia are expressed per natural log-transformed pg/ml higher plasma NfL, GFAP or t-tau. Model 1 adjusted for age and sex. Model 2 additionally adjusted for education level, diabetes status, smoking history, body mass index, total cholesterol-to-HDL cholesterol ratio, use of lipid-modifying medication, systolic blood pressure, and use of antihypertensive medication. Models 1 and 2 represent the total effect, and model 2 + adjustment for WMHV represents the direct effect. Total and direct effect are defined in Supplementary Figure 2.

Abbreviations: NfL, neurofilament light; GFAP, glial fibrillary acidic protein; t-tau, total-tau; WMHV white matter hyperintensity volume.

[a] WMHV expressed per SD percentage of total intracranial volume.

References

1. Rijnhart JJM, Twisk JWR, Eekhout I, Heymans MW. Comparison of logistic-regression based methods for simple mediation analysis with a dichotomous outcome variable. *BMC Med Res Methodol*. 2019;19:19. doi: 10.1186/s12874-018-0654-z
